# St-N, a novel alkaline derivative of stevioside, reverses docetaxel resistance by targeting lysosomes *in vitro* and *in vivo*

**DOI:** 10.1371/journal.pone.0316268

**Published:** 2024-12-27

**Authors:** Yanxia Guo, Shikang Wang, Qun Liu, Yan Dong, Yongqing Liu

**Affiliations:** 1 Department of Central Laboratory, Shandong Provincial Hospital Affiliated to Shandong First Medical University, Jinan, China; 2 Engineering Laboratory of Urinary Organ and Functional Reconstruction of Shandong Province, Shandong Provincial Hospital Affiliated to Shandong First Medical University, Jinan, China; 3 Department of Surgery, Shandong Provincial Hospital Affiliated to Shandong First Medical University, Jinan, China; 4 Department of Pharmacy, The Affiliated Hospital of Shandong University of Traditional Chinese Medicine, Jinan, China; 5 Department of Pharmacy, The Second Hospital, Cheeloo College of Medicine, Shandong University, Jinan, China; BRAC University, BANGLADESH

## Abstract

Drug resistance of cancers remains a major obstacle due to limited therapeutics. Lysosome targeting is an effective method for overcoming drug resistance in cancer cells. St-N (*ent*-13-hydroxy-15-kaurene-19-acid *N*-methylpiperazine ethyl ester) is a novel alkaline stevioside derivative with an amine group. In this study, we found that docetaxel (Doc)-resistant prostate cancer (PCa) cells were sensitive to St-N. Mechanistically, the alkaline characteristic of St-N led to targeting lysosomes, as evidenced by lysosomal swelling and rupture through transmission electron microscopy and Lyso-tracker Red staining. St-N destabilized lysosomal membrane by impairing lysosomal membrane proteins and acid sphingomyelinase. As a result, St-N caused cathepsins to release from the lysosomes into the cytosol, eventually triggering apoptotic and necrotic cell death. Meanwhile, the cytoprotective role of lysosomal activation under docetaxel treatment was interrupted by St-N, leading to significant synergistic cytotoxicity of docetaxel and St-N. In docetaxel-resistant PCa homograft mice, St-N significantly inhibited the growth of RM-1/Doc homografts and enhanced the anticancer effects of docetaxel, but did not show significant toxicity. Taken together, these findings demonstrated that St-N reversed docetaxel resistance *in vitro* and *in vivo* by destabilizing lysosomal membranes to promote cell death, thus providing a strong rationale for applying St-N in docetaxel-resistant PCa.

## Introduction

Despite exciting developments in cancer therapy, acquired drug resistance to most cancer therapeutics remains a major obstacle to the successful treatment of advanced malignancies [1, 2]. Because cancer cells present a very high degree of molecular heterogeneity, therapy-induced selection of a resistant cell subpopulation is almost inevitable, ultimately resulting in drug resistance [3]. Numerous molecular mechanisms that drive therapy-induced drug resistance have been identified, including the activation of ATP-binding cassette drug transporters that trigger drug efflux from cells, overexpression of anti-apoptotic proteins, changes in drug target levels, and/or drug target mutations that result in ineffective treatment, undesirable off-target effects of drug treatments that may activate alternative pathways, and new mechanisms of resistance [1, 4, 5].

Among the various mechanisms of drug resistance, targeting lysosomes in cancer has emerged as an attractive strategy to overcome therapy-mediated resistance [6, 7]. Lysosomes, as key monolayer-membrane subcellular organelles, play a critical role in multiple cellular processes such as post-translational protein maturation, autophagy, receptor degradation, apoptosis, energy metabolism, and cell signaling [7, 8]. Lysosomes in cancer cells are numerous, larger, less stable, and have greater cathepsin activity than lysosomes in normal cells [7, 8].

It is now recognized that lysosomes contribute to drug resistance largely through an ‘off-target’ mechanism in which hydrophobic and weakly basic chemotherapeutic agents are trapped in the acidic environment of lysosomes, sequestering them from their targets [9]. Correspondingly, resistant cells have been found to have an elevated number of lysosomes and the capacity to sequester drugs [9, 10]. Some widely used chemotherapeutic drugs, such as cisplatin, doxorubicin, and docetaxel (Doc), can accumulate in lysosomes, thereby blunting their effects [10–12]. Targeting lysosomes, therefore, holds promise in cancer because it not only induces apoptotic and lysosomal cell death pathways and inhibits cytoprotective autophagy but also reverses drug resistance by limiting the efficiency of sequestering drugs. Recently, several approaches have been developed to overcome drug resistance or exploit lysosomal drug sequestration, including disruption of lysosomal acidification, modulation of acid sphingomyelinase (ASM), and drug-induced lysosomal membrane permeabilization (LMP) [7, 13–15].

Given that basic drugs accumulate more easily in lysosomes, we previously developed a weakly basic compound, *ent*-13-Hydroxy-15-kaurene-19-acid *N*-methylpiperazine ethyl ester (St-N) [16]. St-N is a naturally derived *ent*-kaurane diterpenoid that introduces *N*-methylpiperazine into stevioside, a natural diterpenoid isolated from *Stevia rebaudiana* (Bertoni), without significant anticancer activity [17]. Our previous *in vitro* screening assays showed that St-N exerts potent anticancer effects against hormone-refractory prostate cancer (HRPC). Doc, a widely used taxane antimitotic chemotherapeutic agent, is currently used as first-line chemotherapy for patients with HRPC; however, acquired resistance to Doc remains fatal because of limited effective therapies [4, 18]. Based on the profound effect of lysosomes on cancer drug resistance and the alkaline characteristics of St-N, the present study aimed to test the hypothesis that St-N has a potential therapeutic effect on drug-resistant prostate cancer (PCa) *in vitro* and *in vivo* by targeting lysosomes. We also examined the synergistic activity of combination treatment with St-N and Doc to overcome the survival advantage of drug-resistant cells.

## Materials and methods

### Chemicals and reagents

St-N was synthesized from stevioside by introducing an *N*-methylpiperazine group, and its purity and structure were determined as previously described [16]. Docetaxel was purchased from Aventis Pharma (Dagenham, UK). 3-(4,5-dimethylthiazol-2-yl)-2,5-diphenyl-2H-tetrazolium bromide (MTT) was obtained from Sigma-Aldrich (St. Louis, MO, USA). LysoTracker Red and Hoechst33342 were purchased from the Beyotime Institute of Biotechnology (Beijing, China). E64d, CA074Me, and pepstatin A were purchased from Enzo Life Sciences (Farmingdale, NY, USA). Z-RR-AMC was purchased from EMD Chemicals (Gibbstown, NJ, USA). Annexin V-FITC/PI Apoptosis Detection Kits were purchased from BD Biosciences (Franklin Lakes, NJ, USA). Lactate dehydrogenase (LDH) assay kits were purchased from KeyGen Biotech (Nanjing, China). Amplite^™^ Fluorometric Sphingomyelin Assay Kits were purchased from AAT Bioquest Inc (Pleasanton, CA, USA).

### Cell culture and treatments

Human PCa PC3 cells (The Cell Bank of Chinese Academy of Sciences, Shanghai, China) and Doc-resistant lines PC3/Doc cells were maintained in F12K medium supplemented with 10% fetal bovine serum (HyClone, Logan, UT, USA) and 100 U/mL penicillin and 100 μg/mL streptomycin. Human PCa DU145 cells, murine PCa cell RM-1 cells (The Cell Bank of Chinese Academy of Sciences, Shanghai) and Doc-resistant RM-1/Doc cells were cultured in RPMI-1640 medium supplemented with 10% fetal bovine serum (HyClone) and penicillin and streptomycin. The Doc-resistant cell lines PC3/Doc and RM-1/Doc were selected by growing PC3 and RM-1 cells in increasing concentrations of Doc, and were maintained in medium with 1 nM Doc, as previously described [19]. The human prostate epithelial cell line RWPE-1 (The Cell Bank of Chinese Academy of Sciences) was maintained in Keratinocyte1 medium supplemented with 50 mg/L bovine pituitary extract and 5 μg/L epidermal growth factor (Gibco, Grand Island, NY, USA). All cells were routinely cultured in a humidified incubator with 5% CO_2_ at 37°C.

### Cell viability and cell death assays

After treatment with indicated concentrations of St-N for 48 h, cell proliferation was quantified using the 3-(4,5-dimethylthiazol-2-yl)-2,5-diphenyl-2H-tetrazolium bromide (MTT) colorimetric assay by measuring the light absorbance at 570 nm on a Multiskan microplate reader (Thermo Fisher Scientific, Waltham, MA, USA).

For the cell death assay, cells were exposed to indicated concentrations of St-N for different times. Cell death was assessed using the Annexin V-FITC/PI Apoptosis Detection Kit, and fluorescence was quantified using flow cytometry (CytoFLEX; Beckman Coulter, Brea, CA, USA).

### Transmission electron microscopy (TEM)

TEM was used to analyze the cellular ultrastructure. Cells were fixed with 2.5% glutaraldehyde in 0.1 mmol/L PBS buffer and then incubated with 1% osmium tetroxide. After a dehydration series and embedding in Epon resin, the ultrathin sections were cut and stained with 2% uranyl acetate. Thereafter, samples were visualized using a JEM-1400 transmission electron microscope (JEOL, Tokyo, Japan).

### Lysosomal integrity assay

LysoTracker Red, a red fluorescent dye used to label and track acidic organelles in live cells, was used to assess lysosomal integrity. PC3/Doc cells at various stages after treatment were incubated with LysoTracker at 37°C for 15 min. The fluorescence derived from the aggregated LysoTracker in the acidic compartments was measured using a confocal microscope (Leica TCS SP8; Leica Microsystems, Wetzlar, Germany). Fluorescence was assessed by flow cytometry (CytoFLEX; Beckman Coulter) and was quantified by measuring the geometric mean (GMean) of fluorescence intensity.

### Western blotting assay

Cell lysates were prepared using RIPA buffer for western blotting. The protein concentration was assessed using the BCA protein assay (Beyotime Institute of Biotechnology). The primary antibodies used were as follows: cathepsin B (CTSB; cat. no. 12216-1-AP; Proteintech, Wuhan, China), Microtubule-associated protein 1 light chain 3 beta (LC3B; cat. no. NB100-2220; Novus Biologicals, Littleton, CO, USA), lysosome-associated membrane protein 1 (LAMP1; cat. no. 21997-1-AP; Proteintech), lysosome-associated membrane protein 2 (LAMP2; cat. no. 66301-1-Ig; Proteintech), p62/SQSTM1 (cat. no. sc-28359; Santa Cruz Biotechnology, Dallas, TX, USA), tubulin alpha 1b (TUBA1B; cat. No. 11224-1-AP; Proteintech), cathepsin D (CTSD; cat. No. 21327-1-AP; Proteintech), poly (ADP-ribose) polymerase 1 (PARP1; cat. No. 80174-1-RR; Proteintech), β-actin (ACTB; cat. No. 60008-1-Ig; Proteintech), and glyceraldehyde-3-phosphate dehydrogenase (GAPDH; cat. no. sc-365062; Santa Cruz Biotechnology). GAPDH or ACTB served as protein-loading controls.

### Real-time quantitative PCR (RT-qPCR) analysis

Total RNA was extracted using an RNAiso Plus kit (Takara Bio Inc., Shiga, Japan). cDNA was prepared using a PrimeScript RT Reagent Kit (Takara Bio Inc). RT-qPCR was performed using SYBR Green on an QuantStudio 5 Real-Time PCR System (Thermo Fisher Scientific, Inc.). The primers used to amplify the target genes are listed in S1 Table.

### Subcellular fractionation and CTSB activity assay

The effect of St-N on subcellular fractionation was measured as previously described [20]. The lysed lysosomal and cytosolic fractions were used for the CTSB activity assay. The protein levels of the fractions were quantified using the BCA assay and analyzed by western blotting. CTSB activity was measured after incubating equal amounts of protein (10 μg) with the fluorescent substrate Z-RR-AMC (50 μM; ex. = 365 nm, em. = 449 nm) in 200 μL CTSB reaction buffer for 15 min at 37°C.

### Anticancer efficiency of St-N in vivo

The androgen independent murine RM-1 cells are from C57BL/6 mouse prostate tumors and can be transplanted into C57BL/6 mice to reconstitute the homograft animal models, which is suitable model to simulate human PCa [21, 22]. For the generation of homotransplantations, six-week-old C57BL/6 male mice (Vital River Laboratories, Beijing, China) were inoculated subcutaneously into the right armpit with 1 × 10^5^ RM-1 cells and their paired Doc-resistant RM-1/Doc cells in 0.1 mL of physiological saline to establish Doc-sensitive and Doc-resistant PCa homografts, respectively, as previously described [19, 22]. For the dosage of St-N and Doc, we referred to other lysosomotropic compounds reported previously [10, 23] and our previous study [19]. Mice were housed in a standard environment which was characterized by 12 h light/dark cycle, 22–25°C, and 40–60% humidity with free access to water and chow. All animals were maintained in specific pathogen-free facilities. One week after subcutaneous inoculation, the RM-1 and RM-1/Doc homografts (body weight ~21–22 g) were randomly assigned to four groups (N = 20, n = 5/group for the RM-1 and RM-1/Doc homografts respectively) and treated with vehicle, 5 mg/kg Doc, 30 mg/kg St-N, or a combination of 30 mg/kg St-N and 5 mg/kg Doc. The drugs were intraperitoneally injected every 2 days for 12 days (six times in total). Animal weight and tumor volume were measured every 2 days. Tumor sizes were calculated using the formula 0.5 × L × W^2^ (L = length, W = width). Anaesthetization of mice was conducted with isoflurane and euthanization was performed with CO_2_ followed by cervical dislocation. All animal experiments were approved by the Ethics Committee of the Second Hospital of Shandong University [Approval Number: KYLL-2021(KJ)A-0319] and were conducted accordingly.

### Statistical analysis

Data are presented as mean ± SD. Student’s *t*-test was used to assess the statistical differences between the treated and control groups. Multiple group comparisons were performed using one-way ANOVA followed by Dunnett’s or Tukey’s multiple comparison test. Statistical significance was set at *P* < 0.05, and *P* < 0.001 was considered highly significant.

## Results

### Docetaxel-resistant PCa cells are sensitive to St-N

**Fig 1:**
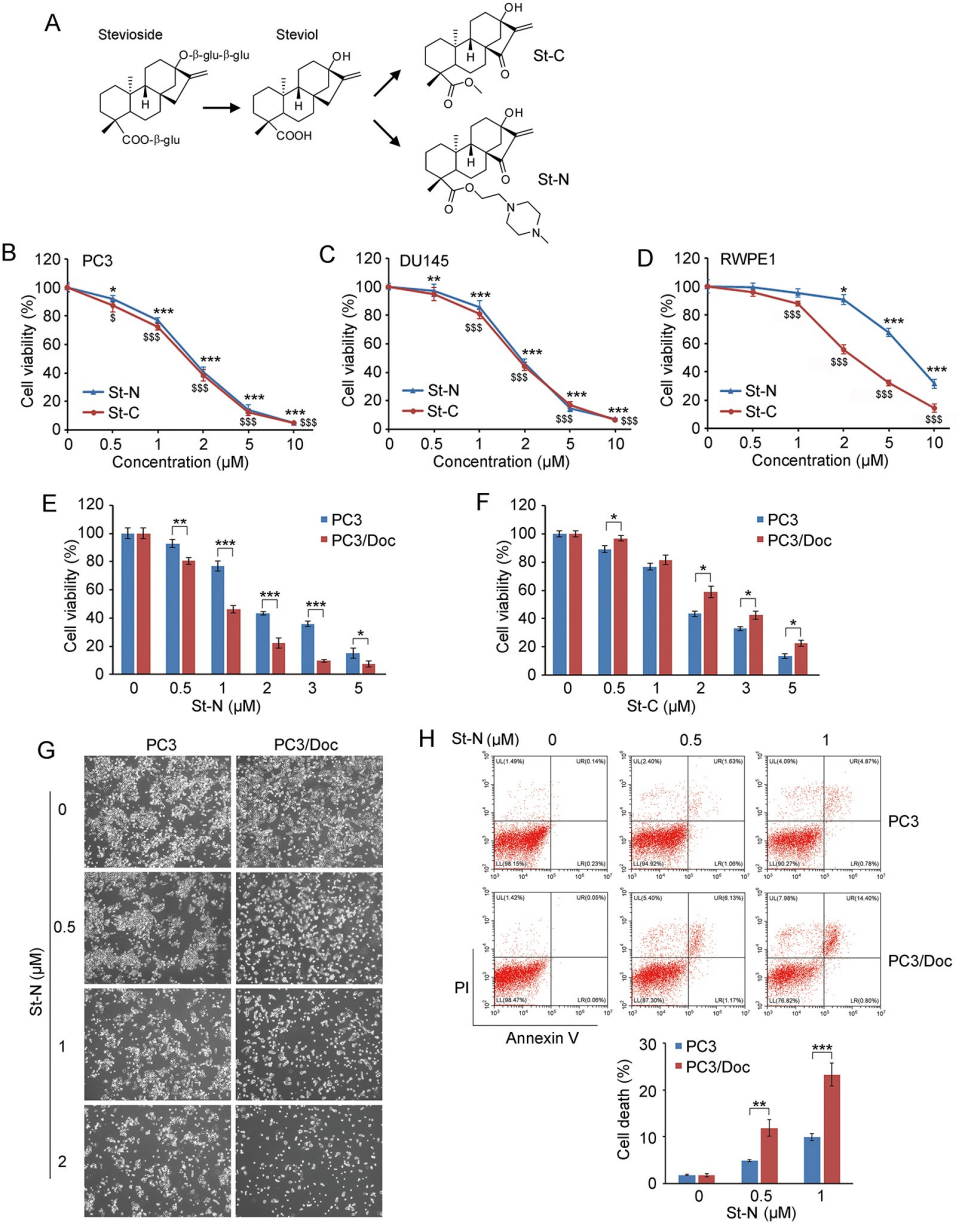
St-N inhibited cell survival in Doc-resistant PCa cells. (A) Chemical structures of St-N and St-C. (B–D) Cell viability in response to St-N and St-C was determined by the MTT assay in PC3 (B), DU145 (C), and RWPE1 (D) cells, respectively. *P < 0.05, **P < 0.01, ***P < 0.001 versus St-N-untreated control group. ^$^*P* < 0.05, ^$$$^*P* < 0.001 versus St-C-untreated control group. (E, F) The inhibitory effects of St-N (E) and St-C (F) on established human Doc-resistant PC3/Doc cells and parental PC3 cells. *P < 0.05, **P < 0.01, ***P < 0.001. In (B–F), viability was determined by the MTT assay after 48 h of treatment with St-N and St-C. (G) The morphological changes after treatment with 0, 0.5, 1, and 2 μM St-N in PC3 and PC3/Doc cells. (H) St-N-induced cell death was examined by Annexin V/PI staining and flow cytometric analysis. PC3/Doc and PC3 cells were exposed to 0, 0.5, and 1 μM St-N for 24 h. **P < 0.01, ***P < 0.001.

We initiated this study to test whether the stevioside derivative St-N (Fig 1A) inhibited cancer cell growth in established Doc-resistant PC3/Doc cells (S1 Fig). We evaluated the efficacy and selectivity of St-N against PC3/Doc cells by comparing St-N with the neutral analog St-C (*ent*-13-hydroxy-15-kaurene-19-acid methyl ester, Fig 1A), in which the alkaline *N*-methylpiperazine of St-N is substituted with a methyl group. The chemical concentrations that induced cell death in 50% of untreated and control cells (IC_50_ values) were calculated. As shown in Fig 1B and 1C, the inhibitory abilities of the two analogs, St-N and St-C, were similar in PC3 and DU145 cells. However, unlike its homolog, St-N was much less cytotoxic to non-neoplastic human benign prostate epithelial RWPE1 cells (Fig 1D), indicating the enhanced selectivity of St-N against cancer cells.

For resistant PC3/Doc cells, St-N and St-C displayed different inhibitory effects. PC3/Doc cells were sensitive to St-N, with a significantly reduced IC_50_ value of ~1 μM compared with ~2 μM in PC3 cells (Fig 1E). In contrast, PC3/Doc cells were more resistant to St-C treatment than PC3 cells (Fig 1F), and the IC_50_ values of St-C were approximately 2 and 3 μM for PC3 and PC3/Doc cells under the same conditions, respectively. Morphological analysis showed that PC3/Doc cells were sensitive to St-N (Fig 1G). Annexin V-FITC/PI staining revealed that St-N caused increased the fraction of apoptosis and necrosis in PC3/Doc compared with PC3 cells (Fig 1H). Taken together, St-N exhibits potent inhibitory effects against chemoresistant PC3/Doc cells and is capable of efficiently inducing cell death, including apoptosis and necrosis.

Analysis of the structures of St-N and St-C revealed that introducing an *N*-methylpiperazine group not only improved the selectivity for cancer cells compared with normal cells but also suppressed resistant cell survival to a greater extent at low doses. Altering the intracellular distribution of a drug is an efficient method to improve both its activity and selectivity. Considering the alkaline characteristics of St-N due to its *N*-methylpiperazine group, we were interested in its lysosomotropic ability for further mechanistic studies.

### St-N disrupts lysosomal membrane stability and leads to LMP as an early event

Recent evidence indicates that therapy-resistant cancer cells have an increased number of lysosomes and elevated cathepsin activity compared with those of sensitive cancer cells. LysoTracker staining revealed that lysosomes in resistant PC3/Doc cells were numerous and larger than those in PC3 cells (S2A Fig), corresponding to the view that drug resistance is linked to the enhancement of lysosomal biogenesis and activity. Moreover, the expression profiles of various genes related to metabolism in lysosomal pathways were particularly altered in Doc-resistant PC3/Doc cells compared with those in PC3 cells (S2B Fig), and Cytoscape analysis showed a noticeable role of the lysosome-related proteins LAMP1 and LAPTM4B in the network (S2C Fig). Additionally, the expression of multiple cathepsins was clearly increased in PC3/Doc compared with that in PC3 cells (S2D and S2E Fig).

**Fig 2:**
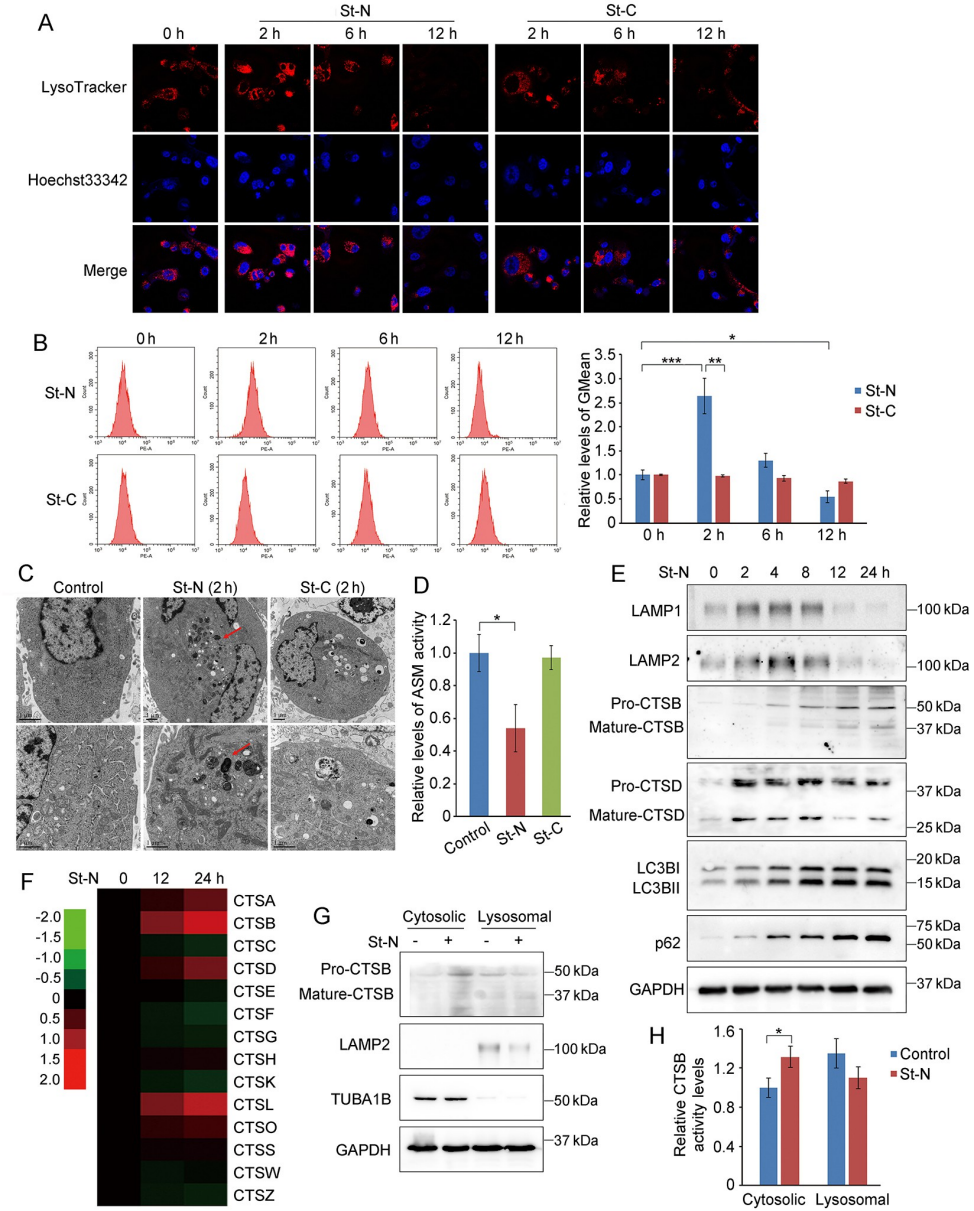
St-N induced lysosomal membrane permeabilization (LMP). (A) Confocal microscopic analysis of lysosomes stained by LysoTracker Red in PC3/Doc cells exposed to 1 μM St-N or St-C for the indicated times. (B) Flow cytometric analysis of lysosomes stained with LysoTracker, and the relative ratio of geometric LysoTracker Red fluorescence. *P < 0.05, **P < 0.01. (C) Electron micrographs showing lysosomal swelling induced by St-N. (D) ASM activities in lysates from the vehicle or St-N-treated PC3/Doc cells. *P < 0.05. (E) Western blotting analysis of LAMP1, LAMP2, CTSB, CTSD, p62, and LC3B in PC3/Doc cells after St-N treatment for different times. (F) Heatmap of the relative mRNA levels [logarithm value (base2)] of various cathepsins, LAMPs, and LC3B in St-N-treated PC3/Doc cells as determined by RT-qPCR. GAPDH served as an internal control. (G) Western blotting analysis of the active CTSB of lysosomal and cytosolic fractions in PC3/Doc cells treated with St-N. LAMP1 served as a lysosomal marker. (H) CTSB activity of lysosomal and cytosolic fractions was measured after subcellular fractionation of St-N-treated PC3/Doc cells. *P < 0.05.

We further examined the effect of St-N on lysosomes. As shown in Fig 2A, lysosomes significantly swelled or fused at a very early stage (2 h) after treatment with 1 μM St-N, after which LysoTracker fluorescence was attenuated and remarkably abolished at 12 h. Similar changes in LysoTracker fluorescence intensity were also noted in St-N-treated PC3/Doc cells through quantitative analysis by flow cytometry (Fig 2B). However, St-C at the same concentration as St-N had no significant effect on LysoTracker fluorescence intensity in PC3/Doc cells (Fig 2A and 2B). In keeping with the above observations, we also observed through transmission electron microscopy that 2 h of treatment with St-N markedly increased lysosome volume, which remained unchanged in St-C-treated PC3/Doc cells (Fig 2C). These results confirmed that St-N profoundly affected lysosomes, which prompted us to further analyze the occurrence of LMP.

The protein and lipid components of the lysosomal membrane play crucial roles in lysosomal integrity and function [7, 24]. We first determined the effect of St-N on ASM activity, which is essential for lysosomal lipid homeostasis to stabilize the lysosomal membrane, and is an important cause of LMP [6]. As shown in Fig 2D, ASM activity was considerably reduced by St-N (~54% versus control at 2 h), supporting the inhibitory activity of St-N in the lysosomal membrane. Additionally, LAMP1 and LAMP2, the most abundant lysosomal membrane proteins important for protein stabilization in the lysosomal membrane, were clearly visible after 2–4 h of treatment with St-N and then largely declined after 12 and 24 h of treatment (Fig 2E), suggesting their involvement in St-N-mediated lysosomal leakage.

The release and activation of cathepsins, a typical event in LMP, is critical for mediating cell death via the lysosomal pathway [7, 25]. As shown in Fig 2E, the active forms of CTSB and CTSD were markedly enhanced at 2–4 h and remained elevated for up to 24 h in St-N-treated PC3/Doc cells. The mRNA expression of various cathepsins was affected by St-N (Fig 2F). Because lysosomal damage impairs the formation of autophagolysosomes and blocks autophagic degradation, we measured whether this event occurred in response to St-N. The results showed that St-N upregulated lipidated LC3B (LC3BII), an autophagosomal marker, and the accumulation of p62/SQSTM1, an autophagy substrate that is degraded in the lysosomes (Fig 2E).

Furthermore, a subcellular fractionation assay showed that St-N increased CTSB in the cytoplasmic fraction, whereas no significant change was observed in lysosomal CTSB (Fig 2G). Correspondingly, CTSB activity in the cytosolic fraction was upregulated by approximately 30% in response to St-N (Fig 2H). Collectively, these data suggest that the alkaline characteristics of St-N lead to the targeting of lysosomes and disruption of lysosomal membrane integrity, in turn contributing to LMP.

### St-N-mediated LMP triggers apoptotic and necrotic cell death

**Fig 3:**
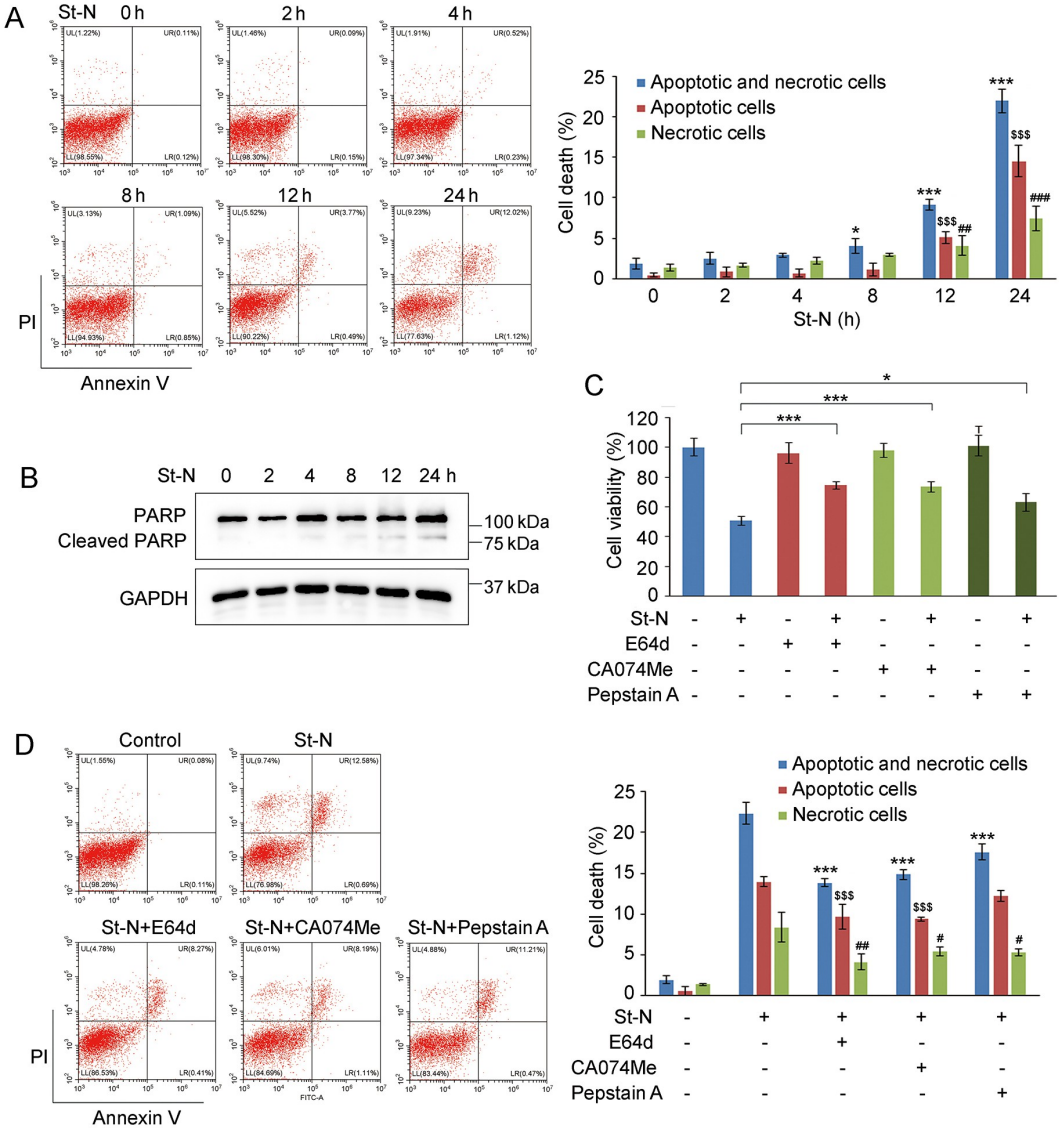
St-N-induced LMP caused cell death. (A) Cell death was measured in PC3/Doc cells exposed to 1 μM St-N for 0, 2, 4, 8, 12, and 24 h by Annexin V/PI staining and flow cytometric analysis. *P < 0.05, ***P < 0.001 versus apoptotic and necrotic cells at 0 h. ^$$$^*P* < 0.001 versus apoptotic cells at 0 h. ^#^*P* < 0.05, ^##^*P* < 0.01 versus necrotic cells at 0 h. (B) The expression of PARP and caspase 3 in St-N-treated PC3/Doc cells at various times. (C) CA074Me, E64d, and pepstatin A attenuated PC3/Doc cytotoxicity induced by St-N, as detected by the MTT assay. Cells were pre-incubated with CA074Me (10 μM), E64d (20 μM), and pepstatin A (20 μM) for 2 h prior to St-N (1 μM) for 48 h. *P < 0.05 and ***P < 0.001. (D) As observed via flow cytometry, CA074-Me, E64d, and pepstatin A attenuated St-N-mediated cell apoptosis. PC3/Doc cells were exposed to CA074Me, E64d, and pepstatin A for 2 h prior to St-N (1 μM) for 24 h. **P < 0.01, ***P < 0.001 versus apoptotic and necrotic cells induced by St-N alone. ^$^*P* < 0.05, ^$$$^*P* < 0.001 versus apoptotic cells induced by St-N alone. ^#^*P* < 0.05 versus necrotic cells induced by St-N alone.

Because the release and activation of cathepsins are critical for mediating cell death via the lysosomal pathway [6, 7], we explored the function of cathepsins in St-N-induced cytotoxicity. Flow cytometry revealed that the fractions of apoptotic and necrotic cells slightly increased at 8 h and significantly increased at 12 and 24 h in response to St-N (Fig 3A). PARP cleavage, a hallmark of apoptosis, was observed at 8 h and became evident after 12 h of St-N treatment (Fig 3B). However, LMP occurred as early as 2 h, as evidenced by lysosomal swelling and cathepsin activation, suggesting that St-N-induced LMP drives apoptosis and necrosis.

We further evaluated the role of St-N-mediated LMP in cell death by determining whether the inhibition of cathepsins attenuated cell death. For these experiments, we preincubated the cells with a cysteine (thiol) proteinase inhibitor (E64d), a cathepsin B inhibitor (CA074Me), and a cathepsin D inhibitor (pepstatin A) before St-N treatment. As shown in Fig 3C, cathepsin inhibition significantly improved cell survival in the presence of St-N. Furthermore, flow cytometry assays demonstrated that E64d, CA074Me, and pepstatin A reduced St-N-triggered cell death from 16.49 to 9.85, 9.69, and 14.78% for apoptotic cells and from 11.7 to 4.44, 5.61, and 7.75% for necrotic cells, respectively (Fig 3D). Therefore, it is reasonable to conclude that St-N-mediated cell death results from lysosomal disruption.

### St-N demonstrates synergistic antitumor effects in combination with Doc in PCa cells

**Fig 4:**
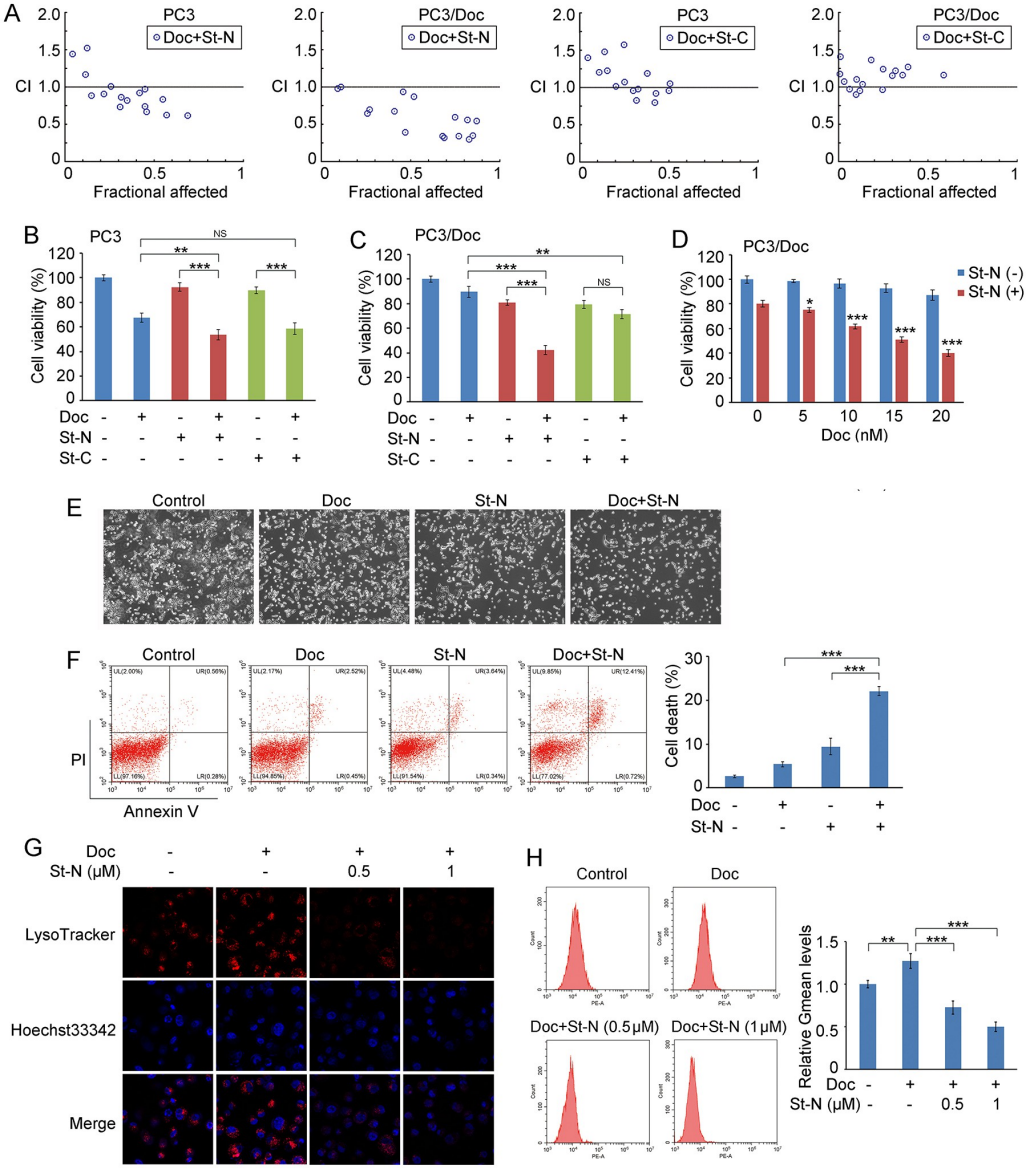
The combined use of St-N and Doc exerted synergistic effect in PCa cells. (A) After treating cells with different concentrations of St-N and Doc for 48 h, the inhibition of cell proliferation was determined by MTT assay, and the combination index (CI) was calculated by CompuSyn software. (B) Cell viability in response to St-N (0.5 μM) or St-C (0.5 μM) combined with Doc (10 nM) for 48 h in PC3 cells. *P < 0.05 and ***P < 0.001. (C) Cell viability in response to St-N (0.5 μM) or St-C (1 μM) combined with Doc (20 nM) for 48 h in PC3/Doc cells. **P < 0.01 and *** *P* < 0.001. (D) Cell viability in response to St-N (0.5 μM) combined with Doc (5–20 nM) for 48 h in PC3/Doc cells. *P < 0.05 and *** *P* < 0.001 compared with single St-N treatment. (E) The morphological changes after co-treatment with St-N and Doc in PC3/Doc cells. (F) St-N (0.5 μM) sensitized PC3/Doc cells to Doc-mediated cell death as measured by flow cytometry. Cells were treated with St-N (0.5 μM), Doc (10 nM), or the combination with for 24 h. ***P < 0.001. (G, H) The effect of the combined use of St-N and Doc on lysosomes observed through LysoTracker staining detected by confocal microscopy (G) or flow cytometry (H). **P < 0.01, ***P < 0.001.

Given the importance of lysosomes in the survival of therapy-resistant cancer cells, we determined whether St-N-induced lysosomal rupture facilitates the effectiveness of Doc in PCa cells. The results in Fig 4A demonstrated that St-N at doses ranging from 0.25–1 μM in combination with Doc predominantly augmented the response of both PC3 and PC3/Doc cells. Notably, St-N produced a stronger synergistic effect in combination with Doc in resistant cells than in PC3 cells, yielding a combination index (CI) of ~0.3 in PC3/Doc cells and ~0.6 in PC3 cells. However, there was no synergistic effect of St-C and Doc in either PC3 or PC3/Doc cells, as shown in Fig 4A. We further analyzed the efficacy of St-N at IC_20_ (0.5 μM) to reverse Doc resistance. In PC3 cells, co-treatment with St-N (0.5 μM) and Doc improved the proliferation inhibition rate (~46%) in comparison with that with single Doc (~32%) or St-N (~7%) treatment in PC3 cells (Fig 4B). Notably, in resistant cells, the combination led to a more pronounced inhibition of cell viability (~60%), with ~10 and ~20% inhibition rates for single Doc and St-N treatments, respectively (Fig 4C); St-N (0.5 μM) in combination with Doc at different concentrations (5–20 nM) also showed synergistic effect in PC3/Doc cells (Fig 4D).

As Doc can induce lysosomal activation to protect against apoptosis [26], St-N has been proposed to sensitize cells to Doc-induced apoptosis by interrupting lysosomal function. Cell morphology shrinkage indicated that co-treatment with St-N and Doc led to increased cell death (Fig 4E). Flow cytometry results also revealed that cell death was remarkably elevated by the combination treatment with St-N and Doc (Fig 4F). Furthermore, LysoTracker staining assays through confocal microscopy (Fig 4G) and flow cytometry (Fig 4H) showed that St-N impaired lysosomal activation by Doc, with ~0.43- and ~0.6-fold decreases in fluorescence intensity versus Doc in response to 0.5 and 1 μM St-N, respectively. These results highlight the antitumor efficacy of St-N in combination with Doc and demonstrate that, in resistant cells, the cytoprotective role of lysosomal activation under Doc treatment is interrupted by St-N.

### Anticancer activity of St-N in Doc-resistant PCa homograft mice

**Fig 5:**
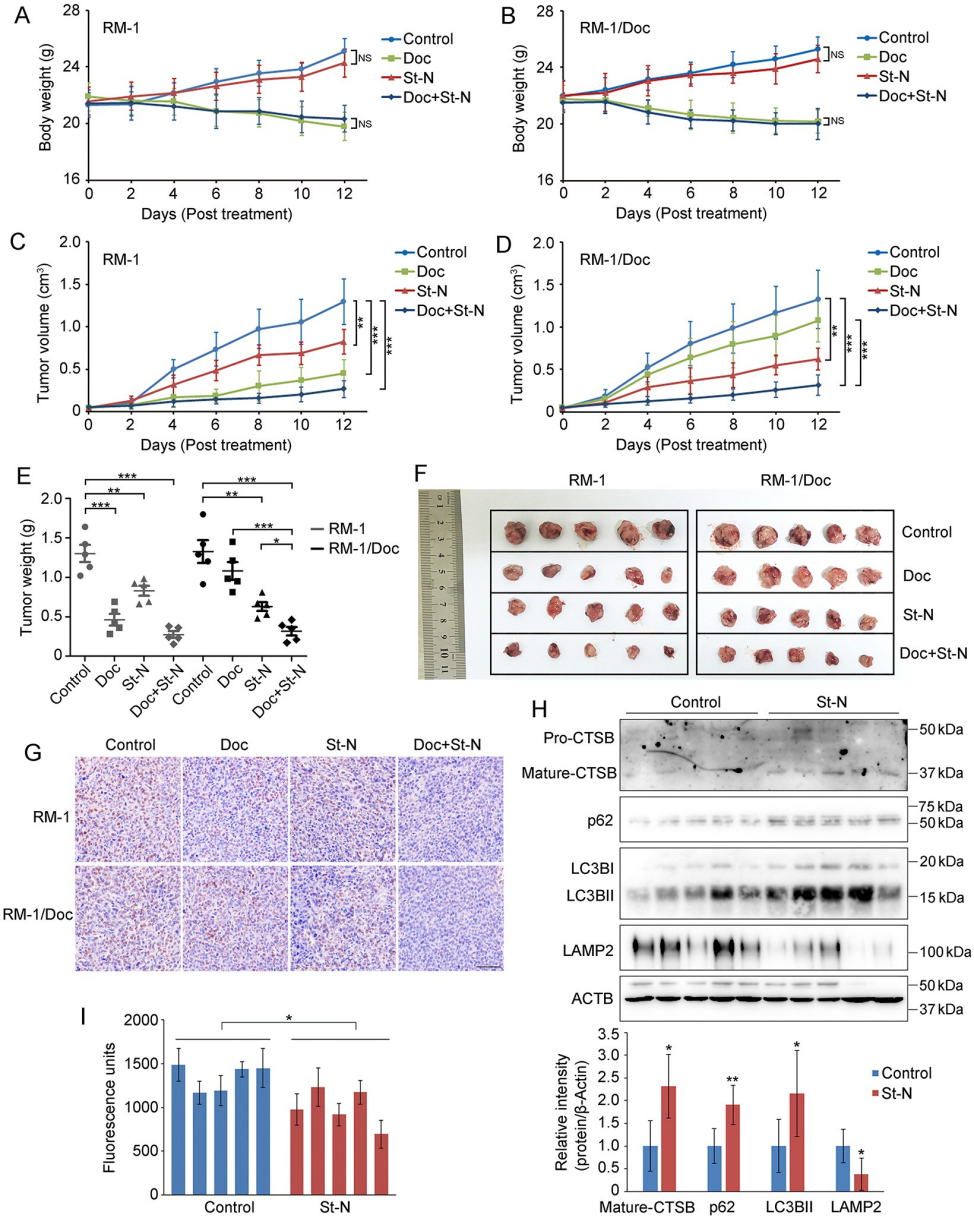
St-N exhibited anticancer activity in Doc-resistant homograft mice. (A–D) Body weights (A, B) and tumor sizes (C, D) were measured every 2 days after the indicated treatment including 5 mg/kg Doc, 30 mg/kg St-N, or a combination of 30 mg/kg St-N and 5 mg/kg Doc. *P < 0.05, **P < 0.01 and ***P < 0.001. (E) Tumor weights were determined at the time of sacrifice. *P < 0.05, **P < 0.01 and ***P < 0.001. (F) Tumors from different groups are shown. (G) Ki67 staining of the indicated treated groups. Scale bar: 50 μm. (H) Effect of St-N on CTSB, p62, LC3B, and LAMP2 expression in RM-1/Doc homografts determined by western blotting. Representative patterns and the corresponding histograms were displayed. *P < 0.05, **P < 0.01 compared with the vehicle control. (I) Effect of St-N on ASM activities in lysates from RM-1/Doc homografts. ***P < 0.001.

Intrigued by the significant effect of St-N on resistant cells, we investigated its potential antitumor efficacy when used alone or in combination with Doc *in vivo*. The Doc-resistant murine RM-1/Doc cell line was also sensitive to St-N (S3 Fig), indicating that it was equivalent to its human counterparts. Thus, RM-1/Doc and its parental RM-1 cell line were used to establish Doc-resistant and Doc-sensitive PCa homografts, respectively, in C57BL/6 mice. As shown in Fig 5A and 5B, the administration of St-N was generally well tolerated by mice without significant loss in body weight, whereas Doc treatment caused an evident reduction in the two groups. Notably, St-N treatment exerted significant inhibitory effects on average tumor volume in both Doc-sensitive RM-1 and Doc-resistant RM-1/Doc homograft groups (Fig 5C and 5D). Correspondingly, tumor weights of St-N-treated RM-1 and RM-1/Doc groups also decreased significantly (0.83 ± 0.14 versus 1.30 ± 0.24 g, *P* < 0.01 in RM-1 group; 0.63 ± 0.13 g versus 1.32 ± 0.32 g, *P* < 0.01 in RM-1/Doc group, Fig 5E and 5F), with the inhibition rates being 36.15 and 52.27%, respectively. For Doc treatment, the growth of RM-1 homografts was significantly inhibited (*P* < 0.001, Fig 5C and 5E); however, no significant reduction was observed in the RM-1/Doc group (*P* > 0.05, Fig 5D and 5E). These results indicated that Doc was effective against PCa, but to a lesser extent against drug-resistant PCa, whereas the inhibitory effect of St-N on chemoresistant tumor growth was clearly evident.

Moreover, the impact of St-N on liver and kidney function was evaluated based on the levels of glutamic-pyruvic transaminase (ALT), glutamate–oxaloacetate transaminase (AST), urea (UREA), and serum creatinine (CREA) in the blood, which were not significantly different (S4 Fig). These results indicated that St-N suppressed tumor growth without causing general toxicity.

We further evaluated the synergistic effects of St-N and Doc on resistant cells *in vivo*. Notably, St-N significantly upregulated the sensitivity of RM-1/Doc homografts to Doc, with an increase in the tumor inhibition rate of ~58% compared with single Doc treatment (*P* < 0.001); the combination also produced a more significant inhibitory effect than St-N alone, increasing the tumor inhibition rate by ~24% (*P* < 0.05) (Fig 5E). The antitumor efficacy of St-N in resistant PCa cells was further determined by Ki67 immunohistochemistry (Fig 5G). Moreover, the combination showed no significant elevation in toxicity compared with that of Doc alone, as evidenced by the almost unchanged mouse body weight (Fig 5A and 5B).

Finally, we examined St-N-induced LMP *in vivo*. Western blotting analysis revealed that the levels of active CTSB, p62, and LC3B increased in St-N-treated RM-1/Doc homografts, further corroborating the occurrence of LMP induced by St-N (Fig 5H). Meanwhile, St-N caused a significant reduction in the expression of LAMP2 (Fig 5H) and ASM activity (*P* < 0.05, versus the vehicle control group; Fig 5I) in RM-1/Doc tumors, indicating changes in lysosomal membranes. Collectively, these data suggest that the lysosomotropic agent St-N is a potential candidate for treating drug-resistant PCa.

## Discussion

Natural products with high structural diversity and interesting biological characteristics are important sources for drug discovery. Our previous study focused on the structural modification of stevioside, a natural diterpenoid. Among its derivatives, St-N, a lysosomotropic derivative synthesized by introducing an amine group, shows greatly improved selectivity for killing cancer cells. In the present study, we identified the potent growth inhibition effect of St-N against Doc-resistant PC3 cells and its synergistic cytotoxicity in combination with Doc *in vitro* and *in vivo*. Furthermore, we demonstrated the mechanisms underlying the St-N-mediated reversal of resistance. The data revealed that St-N destabilized the lysosomal membrane and led to LMP, which in turn triggered cathepsin release and, eventually, cell death. The cytoprotective role of Doc-mediated lysosomal activation was disrupted by St-N, leading to an increase in Doc-induced cell death. This finding is further supported by an *in vivo* study.

In our study, we used MTT assay as an initial screening approach to identify the cytotoxicity of St-N in PC3 and PC3/Doc cells. Although MTT is a widely used method to estimate cell viability and proliferation [27], it can only be used to detect the relative vitality of viable cells, but not for dead or damaged cells. MTT assay is an endpoint determination method that can only provide results at specific time points and cannot monitor the dynamic changes of cells in real time. The results of MTT assay can be also influenced by various conditions, such as acidic pH, polyphenols, and pyruvate analogs [28–30]. Therefore, cytotoxicity detected by MTT may be underestimated or overestimated. The screening results should be carefully confirmed.

Therapy-induced lysosomal activation confers a survival advantage. However, it may produce vulnerabilities that can be exploited using lysosome-targeting agents. Notably, the induction of LMP has emerged as an effective way to kill multidrug-resistant cancer cells. Lysosome-targeting drugs are considered an attractive strategy for re-sensitizing resistant cells to classical chemotherapy [6]. LMP results in the release of lysosomal enzymes, especially cathepsins, from lysosomes into the cytoplasm to initiate cell death. Necroptosis, autophagy, and apoptosis are involved in this outcome, depending on the level of permeabilization [7]. In this process, CTSB, CTSD, and CTSL are the most abundant and ubiquitous cathepsins [31]. They have been demonstrated to be executors of lysosome-mediated cell death by numerous studies [32–34]. The chemical structure of St-N consists of hydrophilic hydroxyl groups and hydrophobic *N*-methylpiperazine ends. Since basic drugs accumulate more easily in lysosomes owing to acidic lysosomal conditions, we hypothesized that St-N targets lysosomes to reverse resistance to Doc. As expected, our data revealed that St-N induced LMP in living cells, as evidenced by lysosomal swelling, changes in lysosomal activity, and cathepsin release. Through time monitoring and pharmacological inhibition using cathepsin inhibitors, we demonstrated that St-N-mediated cathepsin release results in apoptotic and necrotic cell death. Additionally, in line with the view that damage to lysosomes impairs autophagic degradation, St-N represses the degradation of p62 and upregulates its levels in the final step of autophagy. These results strongly suggest that St-N functions as a lysosomotropic agent.

The lysosomal membrane plays a crucial role in maintaining lysosomal function. LMP is caused by changes in the composition of lysosomal membranes. The composition includes membrane lipids and major lysosomal membrane proteins responsible for lysosomal biogenesis and acidification, as well as the transportation of metabolites [7, 24]. The most abundant lysosomal membrane proteins are LAMP1 and LAMP2, representing 50% of all lysosomal membrane proteins. Disruption of these LAMPs decreases the stability of proteins in the lysosomal membrane. The knockdown of either LAMP protein sensitizes cells to lysosomal destabilization [24, 35]. Lipid sphingomyelin is a typical and important component of the cell membrane that supports lysosomal integrity. It is degraded by acid sphingomyelinase (ASM) [36]. ASM inhibition in cancer cells has emerged as another extensively studied approach to induce LMP. Drugs that can reverse drug resistance, such as chloroquine and desipramine, are often lysosomotropic compounds [37, 38]. In the current study, a striking reduction in LAMP1 and LAMP2 levels, and ASM activity was observed in response to St-N. This finding indicated their essential involvement in impairing lysosomal membrane integrity and inducing LMP. Nevertheless, the mode of action of St-N in affecting lysosomal membrane components remains to be further clarified.

Furthermore, our data showed that St-N not only efficiently killed resistant PCa cells but also enhanced the sensitivity of resistant cells to Doc. Resistant cells possess an increased number, volume, and membrane area of lysosomes. The characteristics of lysosomes in resistant cells contribute to drug sequestration in acidic environments and reduce drug effects. Different plasma membrane transporters have been identified as lysosomal transporters. They pump drugs into the lysosomes, resulting in off-target consequences and drug resistance. Thus, inhibiting lysosomal function may be an efficient method for overcoming Doc sequestration. Moreover, it has been reported that Doc activates lysosomal function via enhancement of TFEB activity, and lysosomal activation protects against Doc-mediated apoptosis. Their study suggested that lysosomal inhibition could sensitize cells toward Doc-triggered apoptosis [26]. Accordingly, St-N could impair the cytoprotective role conferred by Doc-mediated lysosomal activation, thus leading to synergistic cytotoxicity. This finding was confirmed by our *in vitro* and *in vivo* results.

## Conclusions

In summary, our results demonstrate that the novel stevioside derivative St-N is a potent lysosomotropic agent that induces LMP. St-N not only overrode the survival advantage of resistant cells but also enhanced the efficacy of Doc by interrupting the cytoprotective role of lysosomal activation *in vitro* and *in vivo*. Since strategies to overcome Doc resistance in PCa remain limited, the present study highlights the potential of targeting lysosomes with St-N to treat Doc-resistant PCa.

## Supporting information

S1 Table The primers for RT-qPCR analysis. (DOCX)

S1 Fig The effect of docetaxel on established human docetaxel-resistant PC3/Doc cells and parental PC3 cells as detected by the MTT assay. (TIF)

S2 Fig (A) Analysis of lysosomes stained by LysoTracker Red and Hoechst 33342 in PC3 and PC3/Doc cells. (B) The relative expression levels [logarithm value (base2)] of various genes related to metabolism in lysosomal pathways in PC3 and PC3/Doc cells. (C) The lysosome related genes with obvious changes in expression were analyzed by Cytoscape software (version: 3.7.1). The results were output to a TSV format file and used Cytoscape for details processing and module analysis. MCODE is a plug-in downloaded from Cytoscape App Storewhich can find closely connected nodes in a complex network based on topology. Therefore, we applied this plug-in to detect critical modules in Protein-Protein Interaction (PPI) network with default parameters. (D) The relative expression levels [logarithm value (base2)] of multiple cathepsins in PC3 and PC3/Doc cells. (E) mRNA levels of cathepsins, LAMPs and LC3B in PC3 and PC3/Doc cells detected by RT-qPCR assay.
(TIF)

S3 Fig The inhibitory effects of St-N on the established murine docetaxel-resistant RM-1/Doc cells compared with parental RM-1 cells. (TIF)

S4 Fig Effect of St-N on ALT, AST, UREA and CREA levels in the blood of RM-1/Doc homograft mice. The levels of ALT, AST, UREA and CREA were detected by using a Seamaty SD1 Dry Biochemistry Analyzer (Seamaty Technology Co., Ltd, Chengdu, China).
(TIF)

S1 Raw image All original images for blots and gels. (PDF)

## References

[1] Dagogo-Jack I, Shaw AT. Tumour heterogeneity and resistance to cancer therapies. Nat Rev Clin Oncol. 2018;15(2):81–94. doi: 10.1038/nrclinonc.2017.166 . 29115304

[2] Sharma P, Hu-Lieskovan S, Wargo JA, Ribas A. Primary, Adaptive, and Acquired Resistance to Cancer Immunotherapy. Cell. 2017;168(4):707–723. doi: 10.1016/j.cell.2017.01.017 . 28187290 PMC5391692

[3] Hanahan D, Weinberg RA. Hallmarks of cancer: the next generation. Cell. 2011;144(5):646–674. doi: 10.1016/j.cell.2011.02.013 . 21376230

[4] Seruga B, Ocana A, Tannock IF. Drug resistance in metastatic castration-resistant prostate cancer. Nat Rev Clin Oncol. 2011;8(1):12–23. doi: 10.1038/nrclinonc.2010.136 . 20859283

[5] Hammerlindl H, Schaider H. Tumor cell-intrinsic phenotypic plasticity facilitates adaptive cellular reprogramming driving acquired drug resistance. J Cell Commun Signal. 2018;12(1):133–141. doi: 10.1007/s12079-017-0435-1 . 29192388 PMC5842196

[6] Groth-Pedersen L, Jaattela M. Combating apoptosis and multidrug resistant cancers by targeting lysosomes. Cancer Lett. 2013;332(2):265–274. doi: 10.1016/j.canlet.2010.05.021 . 20598437

[7] Piao S, Amaravadi RK. Targeting the lysosome in cancer. Ann N Y Acad Sci. 2016;1371(1):45–54. doi: 10.1111/nyas.12953 . 26599426 PMC4879098

[8] Appelqvist H, Waster P, Kagedal K, Ollinger K. The lysosome: from waste bag to potential therapeutic target. J Mol Cell Biol. 2013;5(4):214–226. doi: 10.1093/jmcb/mjt022 . 23918283

[9] Zhitomirsky B, Assaraf YG. Lysosomes as mediators of drug resistance in cancer. Drug Resist Updat. 2016;24:23–33. doi: 10.1016/j.drup.2015.11.004 . 26830313

[10] Niu H, Qian L, Luo Y, Wang F, Zheng H, Gao Y, et al. Targeting of VPS18 by the lysosomotropic agent RDN reverses TFE3-mediated drug resistance. Signal Transduct Target Ther. 2021;6(1):224. doi: 10.1038/s41392-021-00547-x . 34099617 PMC8184988

[11] Herlevsen M, Oxford G, Owens CR, Conaway M, Theodorescu D. Depletion of major vault protein increases doxorubicin sensitivity and nuclear accumulation and disrupts its sequestration in lysosomes. Mol Cancer Ther. 2007;6(6):1804–1813. doi: 10.1158/1535-7163.MCT-06-0372 . 17575109

[12] Huang CP, Fofana M, Chan J, Chang CJ, Howell SB. Copper transporter 2 regulates intracellular copper and sensitivity to cisplatin. Metallomics. 2014;6(3):654–661. doi: 10.1039/c3mt00331k . 24522273 PMC3982597

[13] Le Joncour V, Filppu P, Hyvonen M, Holopainen M, Turunen SP, Sihto H, et al. Vulnerability of invasive glioblastoma cells to lysosomal membrane destabilization. EMBO Mol Med. 2019;11(6). doi: 10.15252/emmm.201809034 . 31068339 PMC6554674

[14] Towers CG, Thorburn A. Targeting the Lysosome for Cancer Therapy. Cancer Discov. 2017;7(11):1218–1220. doi: 10.1158/2159-8290.CD-17-0996 . 29097619 PMC5966281

[15] Seebacher N, Lane DJ, Richardson DR, Jansson PJ. Turning the gun on cancer: Utilizing lysosomal P-glycoprotein as a new strategy to overcome multi-drug resistance. Free Radic Biol Med. 2016;96:432–445. doi: 10.1016/j.freeradbiomed.2016.04.201 . 27154979

[16] Lin Z, Guo Y, Gao Y, Wang S, Wang X, Xie Z, et al. ent-Kaurane Diterpenoids from Chinese Liverworts and Their Antitumor Activities through Michael Addition As Detected in Situ by a Fluorescence Probe. J Med Chem. 2015;58(9):3944–3956. doi: 10.1021/acs.jmedchem.5b00208 . 25856683

[17] Geuns JM. Stevioside. Phytochemistry. 2003;64(5):913–921. doi: 10.1016/s0031-9422(03)00426-6 . 14561506

[18] McKeage K. Docetaxel: a review of its use for the first-line treatment of advanced castration-resistant prostate cancer. Drugs. 2012;72(11):1559–1577. doi: 10.2165/11209660-000000000-00000 . 22818017

[19] Liu YQ, Wang SK, Xu QQ, Yuan HQ, Guo YX, Wang Q, et al. Acetyl-11-keto-beta-boswellic acid suppresses docetaxel-resistant prostate cancer cells in vitro and in vivo by blocking Akt and Stat3 signaling, thus suppressing chemoresistant stem cell-like properties. Acta Pharmacol Sin. 2019;40(5):689–698. doi: 10.1038/s41401-018-0157-9 . 30171201 PMC6786291

[20] Kreuzaler PA, Staniszewska AD, Li W, Omidvar N, Kedjouar B, Turkson J, et al. Stat3 controls lysosomal-mediated cell death in vivo. Nat Cell Biol. 2011;13(3):303–309. doi: 10.1038/ncb2171 . 21336304

[21] Zhang AL, Russell PJ. Paclitaxel suppresses the growth of primary prostate tumours (RM-1) and metastases in the lung in C57BL/6 mice. Cancer Lett. 2006;233(1):185–191. doi: 10.1016/j.canlet.2005.03.051 . 15927363

[22] Xu Q, Liu X, Zhu S, Hu X, Niu H, Zhang X, et al. Hyper-acetylation contributes to the sensitivity of chemo-resistant prostate cancer cells to histone deacetylase inhibitor Trichostatin A. J Cell Mol Med. 2018;22(3):1909–1922. doi: 10.1111/jcmm.13475 . 29327812 PMC5824406

[23] Li L, Sun B, Gao Y, Niu H, Yuan H, Lou H. STAT3 contributes to lysosomal-mediated cell death in a novel derivative of riccardin D-treated breast cancer cells in association with TFEB. Biochem Pharmacol. 2018;150:267–279. doi: 10.1016/j.bcp.2018.02.026 . 29476714

[24] Saftig P, Schroder B, Blanz J. Lysosomal membrane proteins: life between acid and neutral conditions. Biochem Soc Trans. 2010;38(6):1420–1423. doi: 10.1042/BST0381420 . 21118100

[25] Puissant A, Colosetti P, Robert G, Cassuto JP, Raynaud S, Auberger P. Cathepsin B release after imatinib-mediated lysosomal membrane permeabilization triggers BCR-ABL cleavage and elimination of chronic myelogenous leukemia cells. Leukemia. 2010;24(1):115–124. doi: 10.1038/leu.2009.233 . 19924144

[26] Zhang J, Wang J, Wong YK, Sun X, Chen Y, Wang L, et al. Docetaxel enhances lysosomal function through TFEB activation. Cell Death Dis. 2018;9(6):614. doi: 10.1038/s41419-018-0571-4 . 29795139 PMC5966422

[27] Abe K, Matsuki N. Measurement of cellular 3-(4,5-dimethylthiazol-2-yl)-2,5-diphenyltetrazolium bromide (MTT) reduction activity and lactate dehydrogenase release using MTT. Neurosci Res. 2000;38(4):325–329. doi: 10.1016/s0168-0102(00)00188-7 . 11164558

[28] Johno H, Takahashi S, Kitamura M. Influences of acidic conditions on formazan assay: a cautionary note. Appl Biochem Biotechnol. 2010;162(6):1529–1535. doi: 10.1007/s12010-010-8934-z . 20213248

[29] Ganapathy-Kanniappan S, Geschwind JF, Kunjithapatham R, Buijs M, Syed LH, Rao PP, et al. The pyruvic acid analog 3-bromopyruvate interferes with the tetrazolium reagent MTS in the evaluation of cytotoxicity. Assay Drug Dev Technol. 2010;8(2):258–262. doi: 10.1089/adt.2009.0226 . 20085459

[30] Wang S, Yu H, Wickliffe JK. Limitation of the MTT and XTT assays for measuring cell viability due to superoxide formation induced by nano-scale TiO2. Toxicol In Vitro. 2011;25(8):2147–2151. doi: 10.1016/j.tiv.2011.07.007 . 21798338

[31] Rossi A, Deveraux Q, Turk B, Sali A. Comprehensive search for cysteine cathepsins in the human genome. Biol Chem. 2004;385(5):363–372. doi: 10.1515/BC.2004.040 . 15195995

[32] Foghsgaard L, Wissing D, Mauch D, Lademann U, Bastholm L, Boes M, et al. Cathepsin B acts as a dominant execution protease in tumor cell apoptosis induced by tumor necrosis factor. J Cell Biol. 2001;153(5):999–1010. doi: 10.1083/jcb.153.5.999 . 11381085 PMC2174340

[33] Bidere N, Lorenzo HK, Carmona S, Laforge M, Harper F, Dumont C, et al. Cathepsin D triggers Bax activation, resulting in selective apoptosis-inducing factor (AIF) relocation in T lymphocytes entering the early commitment phase to apoptosis. J Biol Chem. 2003;278(33):31401–31411. doi: 10.1074/jbc.M301911200 . 12782632

[34] Conus S, Perozzo R, Reinheckel T, Peters C, Scapozza L, Yousefi S, et al. Caspase-8 is activated by cathepsin D initiating neutrophil apoptosis during the resolution of inflammation. J Exp Med. 2008;205(3):685–698. doi: 10.1084/jem.20072152 . 18299403 PMC2275389

[35] Eskelinen EL. Roles of LAMP-1 and LAMP-2 in lysosome biogenesis and autophagy. Mol Aspects Med. 2006;27(5–6):495–502. doi: 10.1016/j.mam.2006.08.005 . 16973206

[36] Saftig P, Sandhoff K. Cancer: Killing from the inside. Nature. 2013;502(7471):312–313. doi: 10.1038/nature12692 . 24089207

[37] De Milito A, Fais S. Proton pump inhibitors may reduce tumour resistance. Expert Opin Pharmacother. 2005;6(7):1049–1054. doi: 10.1517/14656566.6.7.1049 . 15957961

[38] Shiraishi N, Akiyama S, Kobayashi M, Kuwano M. Lysosomotropic agents reverse multiple drug resistance in human cancer cells. Cancer Lett. 1986;30(3):251–259. doi: 10.1016/0304-3835(86)90049-2 . 3697945

