# Supplementary material for: St-N, a novel alkaline derivative of stevioside, reverses docetaxel resistance by targeting lysosomes in vitro and in vivo
Source: PLoS One. 2024 Dec 27;19(12):e0316268. doi: 10.1371/journal.pone.0316268 (PMC11676526; doi:10.1371/journal.pone.0316268)
Supplement: S1 Table — (DOCX) [file pone.0316268.s001.docx]

**S1 Table.**

The primers for RT-qPCR analysis

| Gene | Forward | Reverse |
| --- | --- | --- |
| LAMP1 | ACGTTACAGCGTCCAGCTCAT | TCTTTGGAGCTCGCATTGG |
| LAMP2 | TGGCAATGATACTTGTCTGCTG | ACGGAGCCATTAACCAAATACAT |
| CTSA | GTCGCCCAGAGCAATTTTGAG | TCTCCCCGGTCAGGAAAAGTT |
| CTSB | AGTGGAGAATGGCACACCCTA | AAGAAGCCATTGTCACCCCA |
| CTSC | CGATGTCAACTGCTCGGTTAT | AAGGCAAACCACTTGTAGTCATT |
| CTSD | TGCTCAAGAACTACATGGACGC | CGAAGACGACTGTGAAGCACT |
| CTSE | AGGCATCCGTCCCTCAAGAA | CCTTGGCACTCTGGTCCATTG |
| CTSF | AGAGAGGCCCAATCTCCGT | GCATGGTCAATGAGCCAAGG |
| CTSG | ACATGGCGTATCTTCAGATCCA | GCGCCCAGGGTGACATTTAT |
| CTSH | CAAGTCATGGATGTCTAAGCACC | CATTGTTGTGGGCGTTTATCTTC |
| CTSK | GCAGAAGAACCGGGGTATTGA | GAAGGAGGTCAGGCTTGCAT |
| CTSL | CTTTTGCCTGGGAATTGCCTC | CATCGCCTTCCACTTGGTC |
| CTSO | GCCGTTAAGATTTGACTGGAGG | GCTTCCCCTTTATTGCATAAGCA |
| CTSS | TGACAACGGCTTTCCAGTACA | GGCAGCACGATATTTTGAGTCAT |
| CTSW | AGATCCAGTTCAACCGGAGTT | TGTGAGGTCACTGAATGGAGT |
| CTSZ | ACCAATGTGGGACATGCAATG | TTTGCGTAGATTTCTGCCATCA |
| LC3B | AAGGCGCTTACAGCTCAATG | CTGGGAGGCATAGACCATGT |
| ACTB | CATGTACGTTGCTATCCAGGC | CTCCTTAATGTCACGCACGAT |
| GAPDH | GGAGCGAGATCCCTCCAAAAT | GGCTGTTGTCATACTTCTCATGG |
