# Supplementary figures and images for: St-N, a novel alkaline derivative of stevioside, reverses docetaxel resistance by targeting lysosomes in vitro and in vivo
Source: PLoS One. 2024 Dec 27;19(12):e0316268. doi: 10.1371/journal.pone.0316268 (PMC11676526; doi:10.1371/journal.pone.0316268)

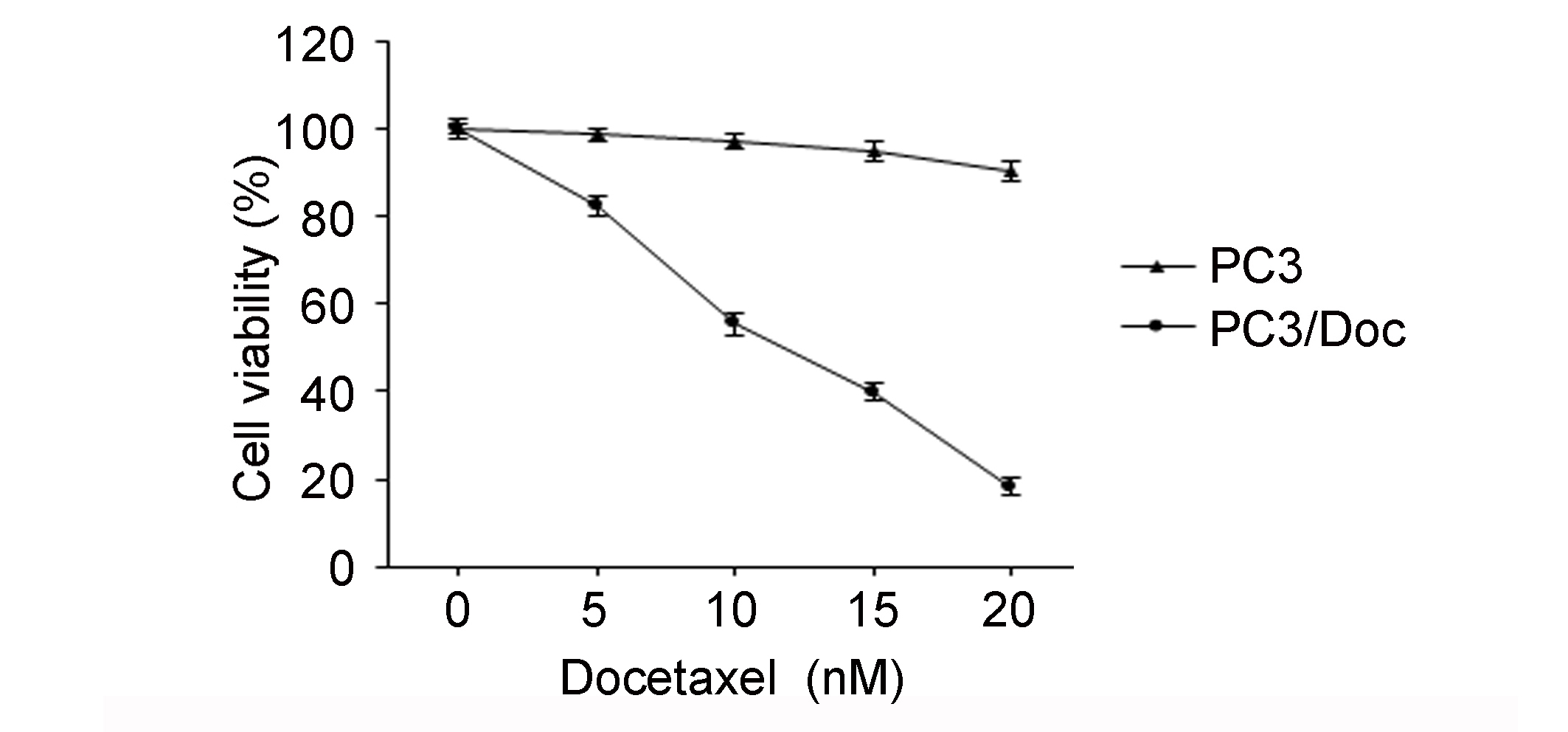

Supplement: S1 Fig — (TIF) [file pone.0316268.s002.tif]

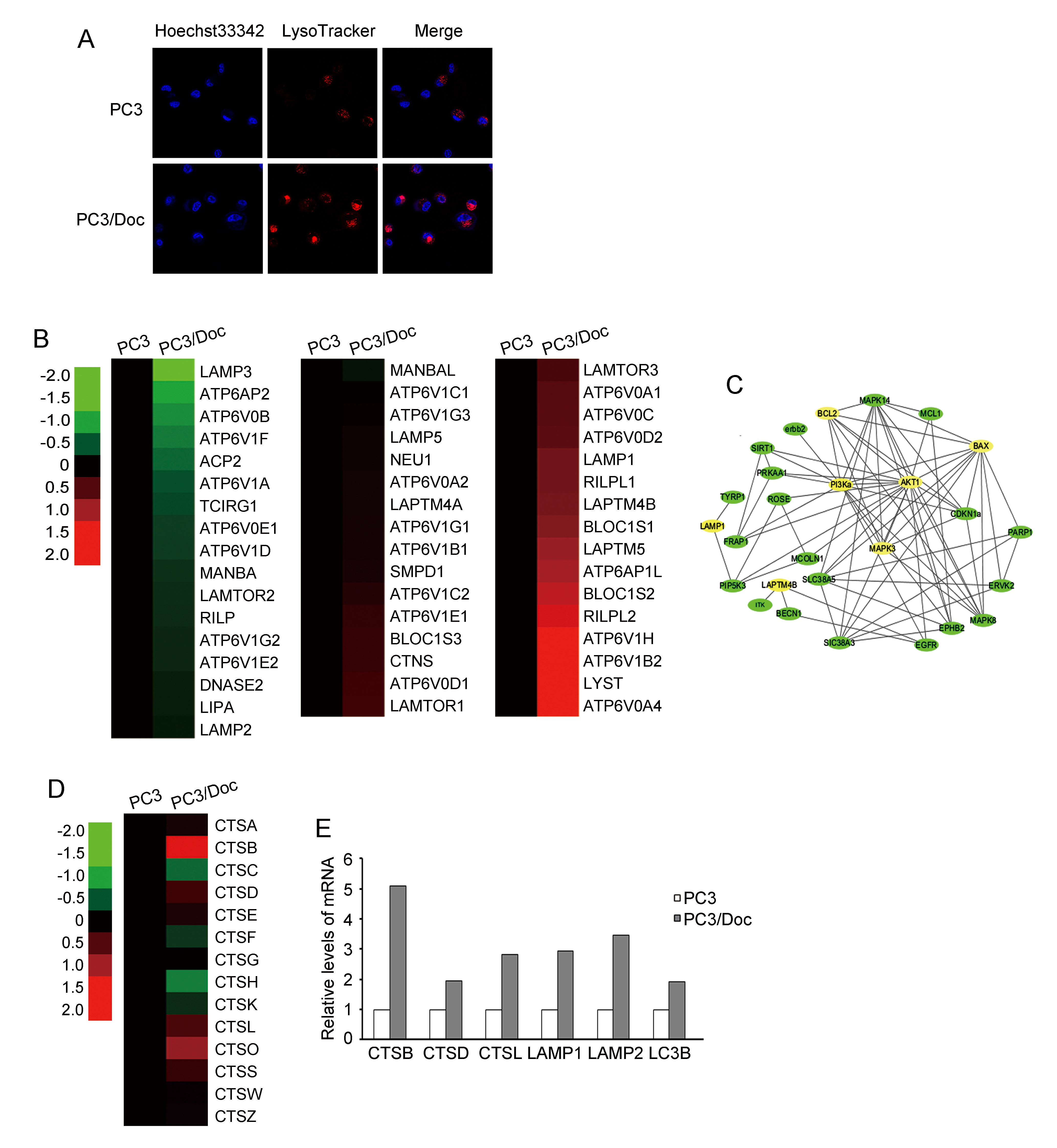

Supplement: S2 Fig — (A) Analysis of lysosomes stained by LysoTracker Red and Hoechst 33342 in PC3 and PC3/Doc cells. (B) The relative expression levels [logarithm value (base2)] of various genes related to metabolism in lysosomal pathways in PC3 and PC3/Doc cells. (C) The lysosome related genes with obvious changes in expression were analyzed by Cytoscape software (version: 3.7.1). The results were output to a TSV format file and used Cytoscape for details processing and module analysis. MCODE is a plug-in downloaded from Cytoscape App Storewhich can find closely connected nodes in a complex network based on topology. Therefore, we applied this plug-in to detect critical modules in Protein-Protein Interaction (PPI) network with default parameters. (D) The relative expression levels [logarithm value (base2)] of multiple cathepsins in PC3 and PC3/Doc cells. (E) mRNA levels of cathepsins, LAMPs and LC3B in PC3 and PC3/Doc cells detected by RT-qPCR assay. (TIF) [file pone.0316268.s003.tif]

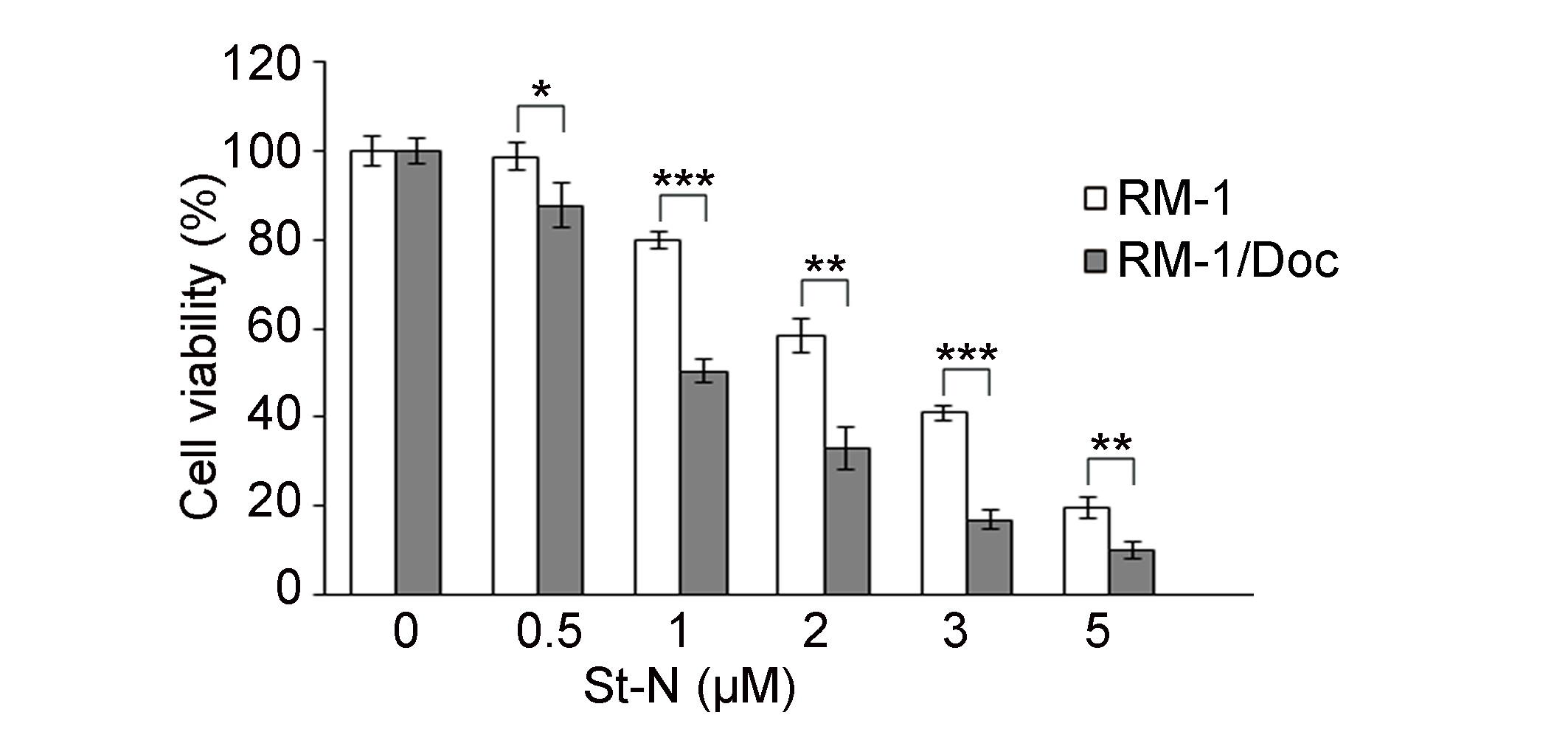

Supplement: S3 Fig — (TIF) [file pone.0316268.s004.tif]

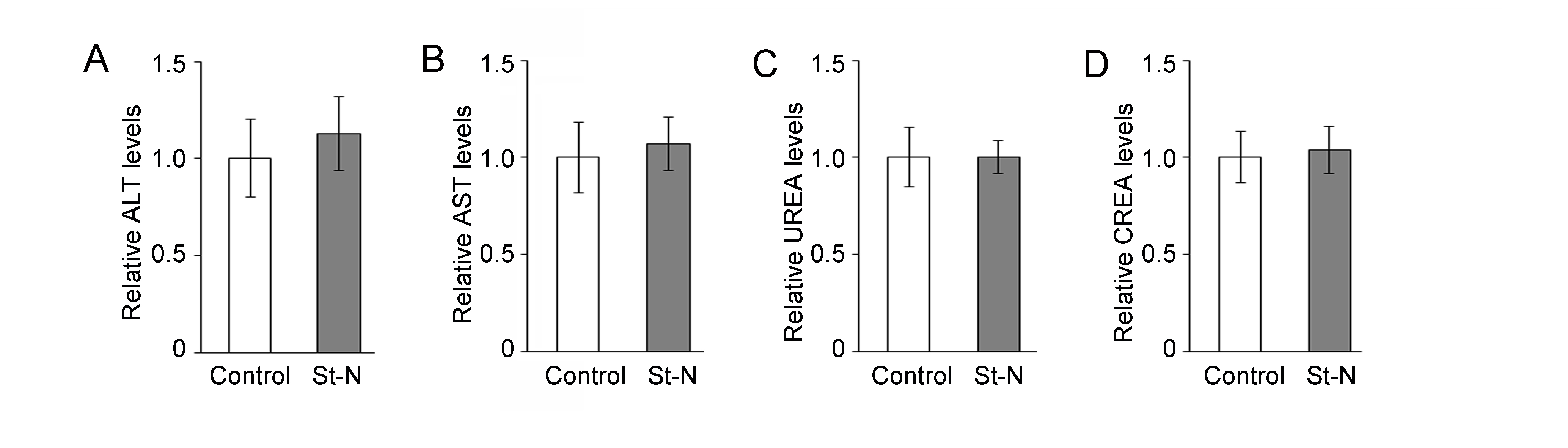

Supplement: S4 Fig — The levels of ALT, AST, UREA and CREA were detected by using a Seamaty SD1 Dry Biochemistry Analyzer (Seamaty Technology Co., Ltd, Chengdu, China). (TIF) [file pone.0316268.s005.tif]
