# Supplementary material for: St-N, a novel alkaline derivative of stevioside, reverses docetaxel resistance by targeting lysosomes in vitro and in vivo
Source: PLoS One. 2024 Dec 27;19(12):e0316268. doi: 10.1371/journal.pone.0316268 (PMC11676526; doi:10.1371/journal.pone.0316268)

All original images for blots were captured by Amersham ImageQuant™ 800 (Cytiva, USA) as shown below.

The panel for Fig 2E LAMP1 was generated from this original image. (The outline highlights which part of the image is included in the Figure)

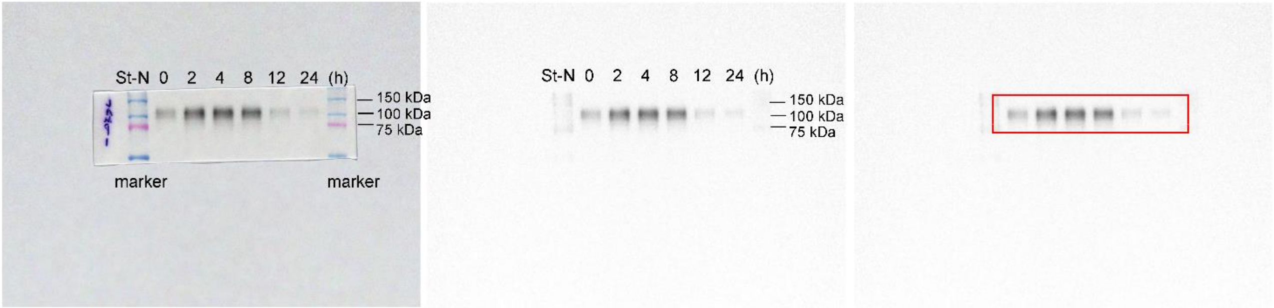

The panel for Fig 2E LAMP2 was generated from this original image. (The outline highlights which part of the image is included in the Figure)

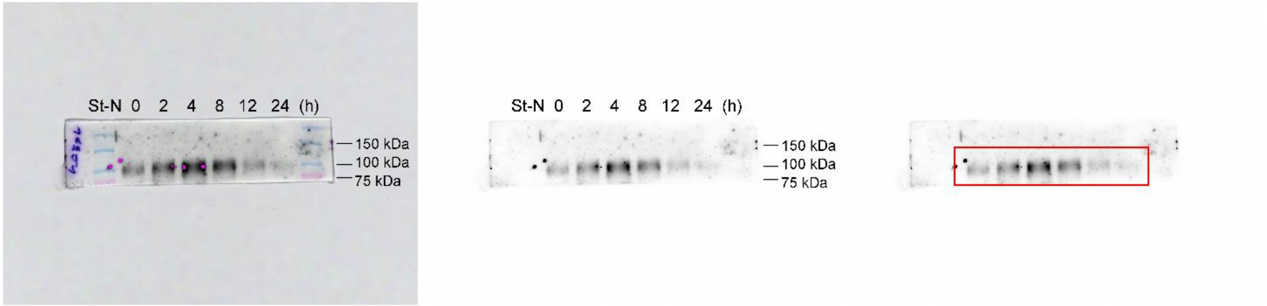

The panel for Fig 2E CTSB was generated from this original image. (The outline highlights which part of the image is included in the Figure)

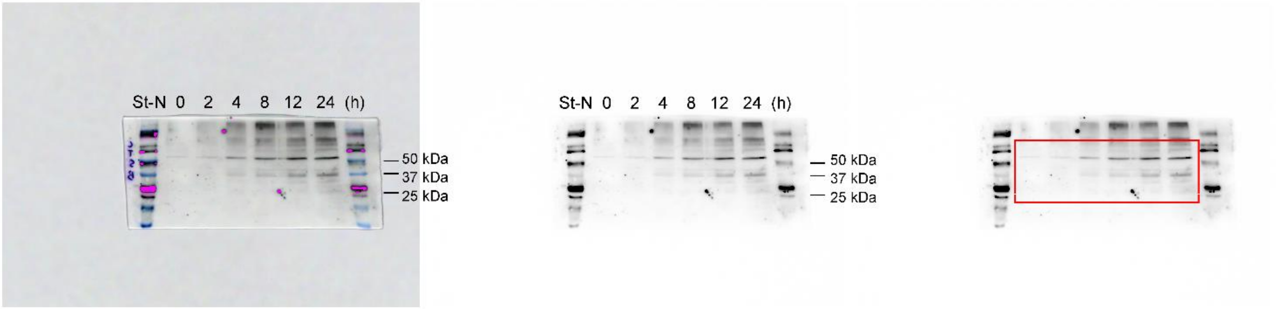

The panel for Fig 2E CTSD was generated from this original image. (The outline highlights which part of the image is included in the Figure)

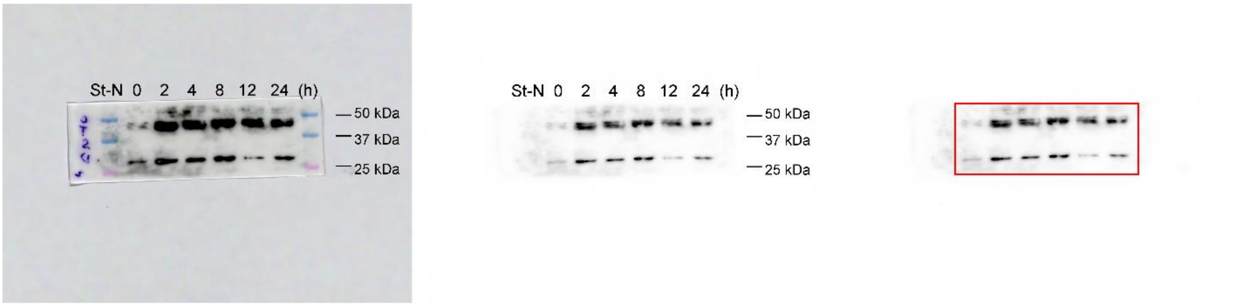

The panel for Fig 2E LC3B was generated from this original image. (The outline highlights which part of the image is included in the Figure)

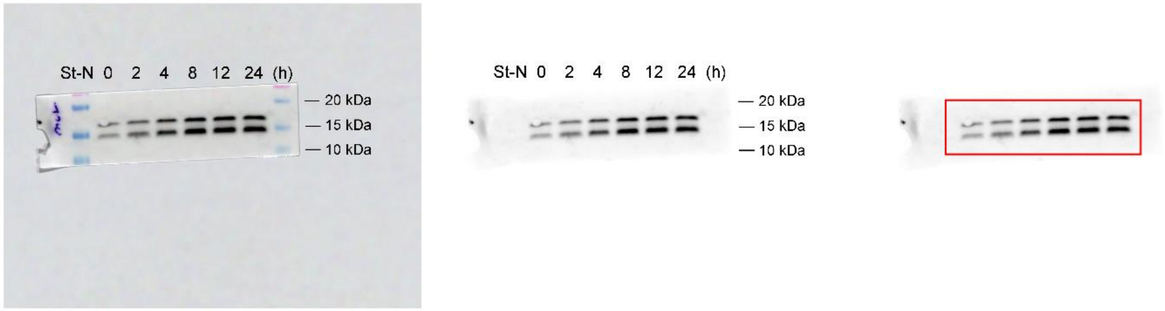

The panel for Fig 2E p62 was generated from this original image. (The outline highlights which part of the image is included in the Figure)

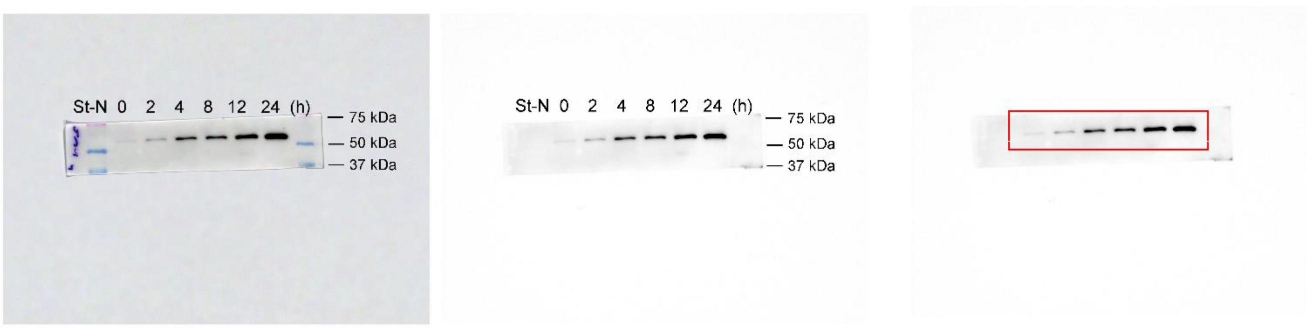

The panel for Fig 2E GAPDH was generated from this original image. (The outline highlights which part of the image is included in the Figure)

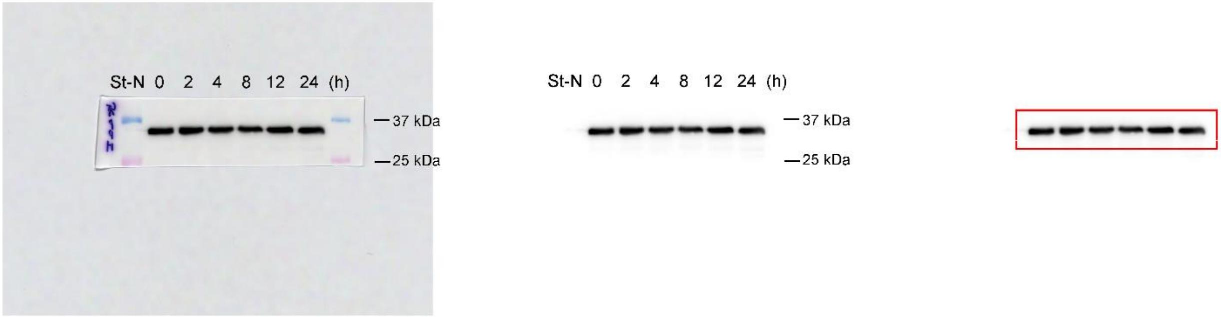

The panel for Fig 2G CTSB was generated from this original image. (The outline highlights which part of the image is included in the Figure)

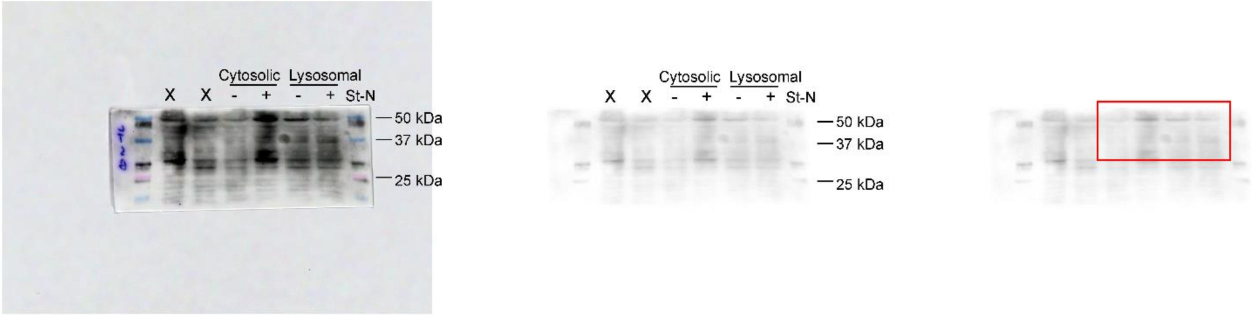

The panel for Fig 2G LAMP2 was generated from this original image. (The outline highlights which part of the image is included in the Figure)

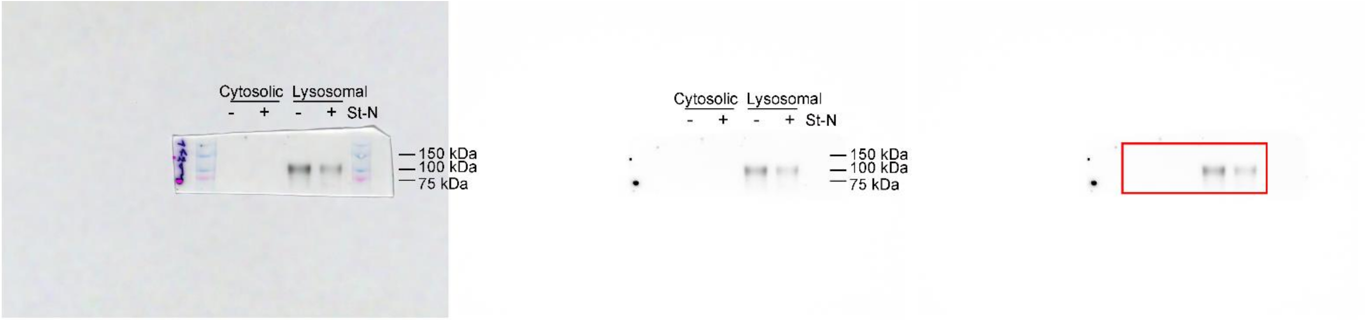

The panel for Fig 2G TUBA1B was generated from this original image. (The outline highlights which part of the image is included in the Figure)

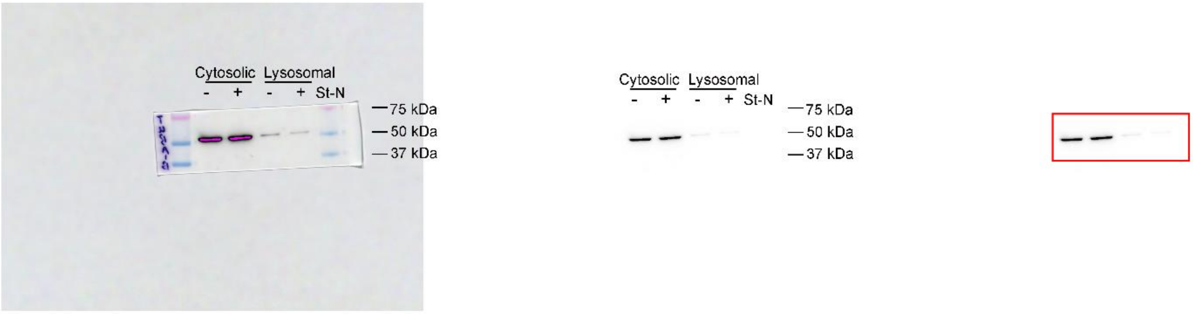

The panel for Fig 2G GAPDH was generated from this original image. (The outline highlights which part of the image is included in the Figure)

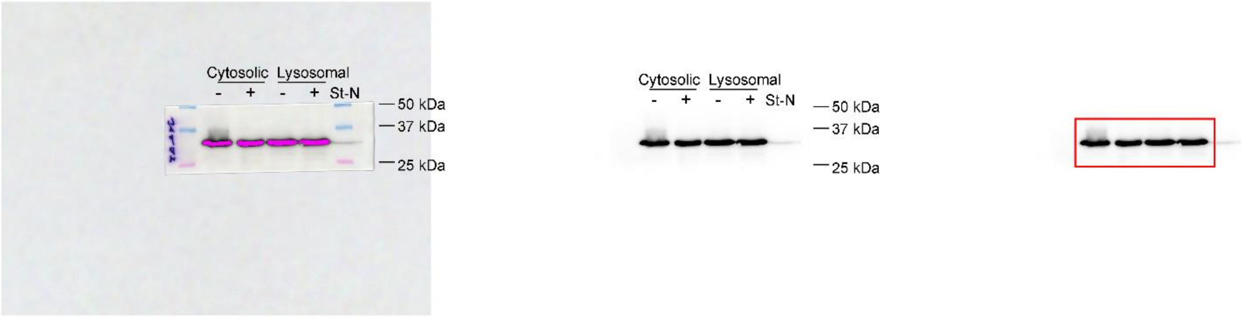

The panel for Fig 3B PARP was generated from this original image. (The outline highlights which part of the image is included in the Figure)

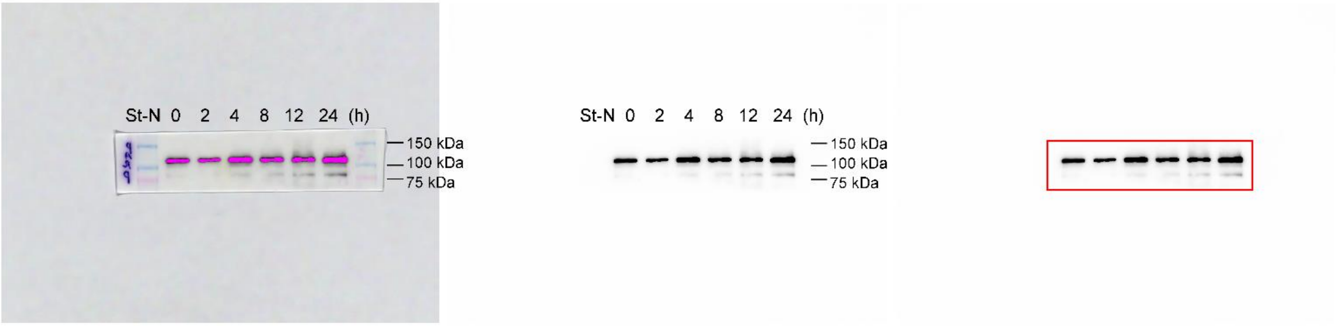

The panel for Fig 3B GAPDH was generated from this original image. (The outline highlights which part of the image is included in the Figure)

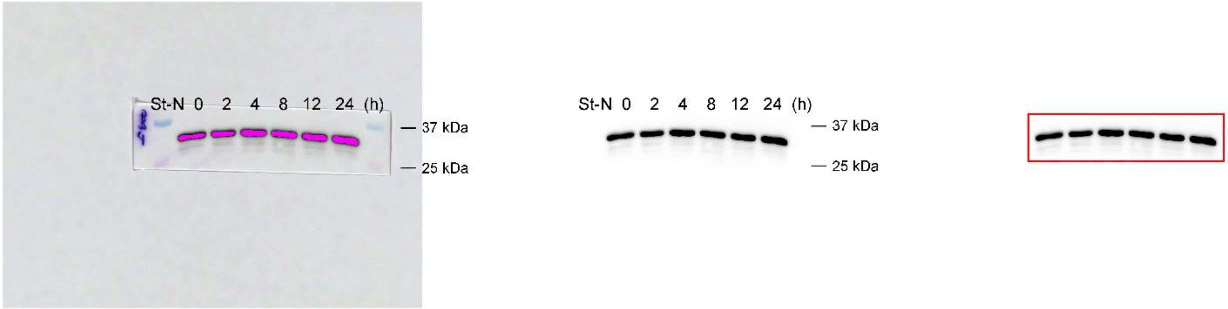

The panel for Fig 5H CTSB was generated from this original image. (The outline highlights which part of the image is included in the Figure)

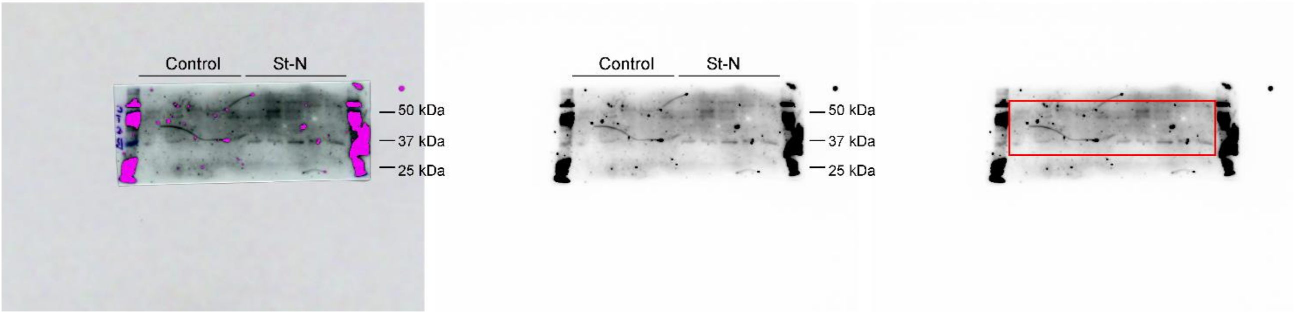

The panel for Fig 5H LAMP2 was generated from this original image. (The outline highlights which part of the image is included in the Figure)

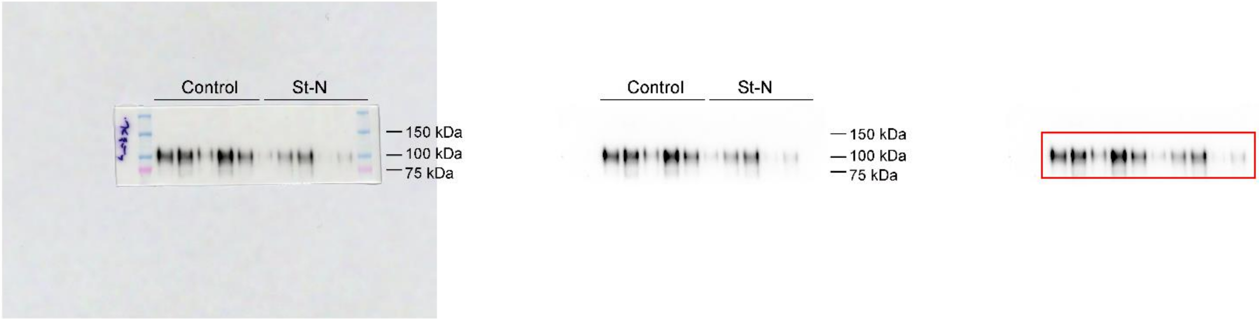

The panel for Fig 5H p62 was generated from this original image. (The outline highlights which part of the image is included in the Figure)

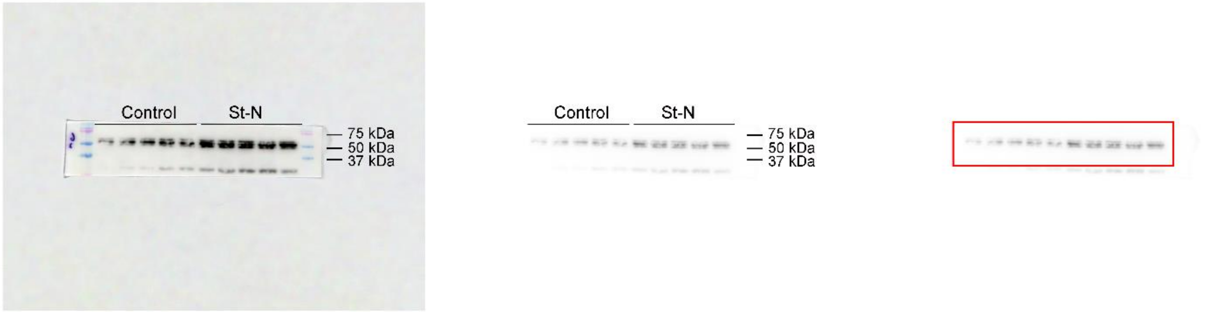

The panel for Fig 5H LC3B was generated from this original image. (The outline highlights which part of the image is included in the Figure)

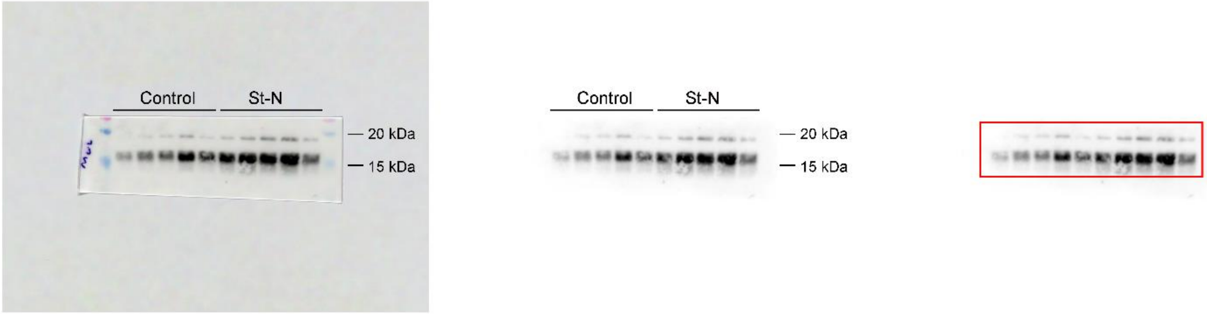

The panel for Fig 5H ACTB was generated from this original image. (The outline highlights which part of the image is included in the Figure)

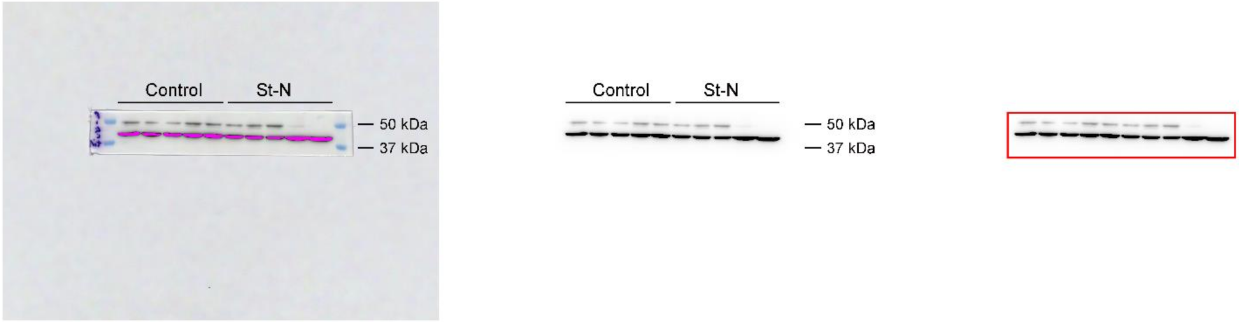

Supplement: S1 Raw image — (PDF) [file pone.0316268.s006.pdf]
